# NMR as a Discovery Tool: Exploration of Industrial Effluents Discharged Into the Environment

**DOI:** 10.1002/mrc.5527

**Published:** 2025-05-13

**Authors:** Kiera Ronda, Jeremy Gauthier, Khanisha Singaravadivel, Peter M. Costa, Katelyn Downey, William W. Wolff, Daniel H. Lysak, Jacob Pellizzari, Owen Vander Meulen, Katrina Steiner, Amy Jenne, Monica Bastawrous, Zainab Ng, Agnes Haber, Benjamin Goerling, Venita Busse, Falko Busse, Colin Elliot, Scott Mabury, Mohamed Ateia, Derek C. G. Muir, Robert J. Letcher, Krish Krishnamurthy, Sonya Kleywegt, Karl J. Jobst, Myrna J. Simpson, Andre J. Simpson

**Affiliations:** ^1^ Environmental NMR Centre, Department of Physical and Environmental Sciences University of Toronto Scarborough Toronto ON Canada; ^2^ Department of Chemistry University of Toronto Toronto ON Canada; ^3^ Bruker Biospin GmbH Ettlingen Germany; ^4^ Bruker Switzerland AG Faellanden Switzerland; ^5^ Department of Chemical and Biomolecular Engineering Rice University Houston Texas USA; ^6^ Canada Centre for Inland Waters, Environment and Climate Change Canada Burlington ON Canada; ^7^ Ecotoxicological and Wildlife Health Division, Environment and Climate Change Canada, National Wildlife Research Centre Carleton University Ottawa ON Canada; ^8^ Chempacker LLC San Jose California USA; ^9^ Technical Assessment and Standards Development Branch Ontario Ministry of the Environment, Conservation and Parks Toronto ON Canada; ^10^ Department of Chemistry Memorial University of Newfoundland St. John's NL Canada

**Keywords:** ^13^C, ^19^F, ^1^H, compound discovery, environmental, industrial effluents, mixture analysis, NMR spectroscopy, novel pollutants, structure elucidation

## Abstract

NMR provides unprecedented molecular information, urgently needed by environmental researchers and policy makers. However, NMR is underutilized in environmental sciences due to the lack of available technologies, limited environmental‐specific training opportunities, and easy‐to‐use workflows. NMR has considerable potential as a discovery tool for novel pollutants, and by‐products, exemplified by the recent discovery of the degradation by‐product of a rubber additive, 6PPD‐quinone, now considered one of the most toxic compounds presently known. This work represents a proof‐of‐concept case study highlighting the use of NMR to profile effluents from 38 industries across Ontario, Canada. Wastewater effluents from various industrial sectors were analyzed using several 1D and 2D ^1^H/^13^C NMR and ^19^F experiments and were screened both unconcentrated and after lyophilization. Common species could be identified using human metabolic NMR databases, but environmental‐specific NMR databases desperately need further development. An example of manually identifying unusual NMR signatures is included; these resulted from phosphinic and phosphonic acids originating from the electroplating industry, for which the environmental impacts are not well understood. Basic ^1^H NMR quantification is performed using ERETIC, while an optimized approach combining relaxation agents and steady‐state‐free‐precession ^19^F NMR, to reduce detection limits (at 500 MHz) to sub‐ppb (< 1 μg/L) in under 15 min, is demonstrated. The future potential of benchtop NMR (80 MHz) is also considered. This paper represents a guide to others interested in applying NMR spectroscopy to environmental media and demonstrates the potential of NMR as a complementary tool to assist MS in environmental pollutant and by‐product discovery.

## Graphical abstract

A proof‐of‐concept case study highlighting the use of NMR to profile effluents from 38 industries across Ontario, Canada: a guide to those interested in applying NMR spectroscopy to environmental media and exploring its potential as a discovery tool.

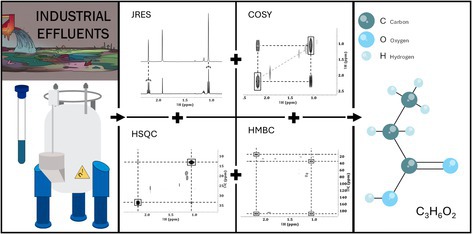

## 1 Introduction

Nuclear magnetic resonance (NMR) spectroscopy is nondestructive and can be applied to unaltered samples such as soils, water, or air particles [1], or to study molecular responses in vivo [2]. Additionally, NMR provides unprecedented molecular information on complex systems due to its unique ability to spectroscopically solve the structures of new pollutants/toxins and understand molecular binding (noncovalent interactions and associations). However, the limited use of this invaluable technique in environmental research has resulted in a heavy reliance on established mass spectrometry (MS) libraries. Consequently, a strong bias toward monitoring previously identified compounds has developed, leaving many novel and potentially hazardous compounds unidentified for years. For example, in 1976, various organofluorine compounds were identified in human blood by ^19^F NMR [3, 4], a discovery that was not confirmed by MS until several decades later. As of now, fluorinated compounds are routinely detected in human tissue at concentrations that far exceed those known to be harmful to human health [5]. Another prime example was the mystery of an unknown stressor responsible for killing up to 90% of the wild coho salmon across the US west coast. Ultimately, in collaboration with the University of Washington and the Environmental Protection Agency (EPA), the Environmental NMR Centre (University of Toronto) identified the structure of this compound using NMR. The compound was identified as a completely novel chemical known as 6PPD‐quinone; the ozonation product of the major tire additive 6PPD.The work, co‐published in 2021 in *Science* [6] has already had considerable impact, with 6PPD now under review for a global ban. 6PPD‐quinone is now found ubiquitously in the environment [7], and in air particle fractions that enter human lungs [8]. Recently, it has been closely linked to numerous respiratory diseases in humans [9]. And recent work has found that as little as 4 weeks of exposure can cause multiple organ injury in mice [10]. Without NMR, 6PPD‐quinone, which is now considered one of the most toxic chemicals ever found, would still be *undiscovered*.

In 2020, a comprehensive analysis of chemical inventories from across the world found that there are more than 350,000 chemicals and chemical mixtures produced globally, of which nearly 70,000 have either unknown or confidential identities. Notably, these inventories do not account for the millions of potential precursor and transformation products that may arise from these products [11]. This represents a massive knowledge gap in terms of the identities of the chemical pollutants that may be inadvertently released into the environment. MS‐based approaches are routinely employed for monitoring chemical pollutants due to the exceptional detection limits of this technique, but MS is limited in its ability to confidently assign novel structures and quantify unknowns in cases where standards are not readily available. However, NMR maps out the connectivity of bonds within a molecule, making it ideal for solving structures. Additionally, when run using quantitative conditions, NMR sees all nuclei/molecules, equally such that electronic referencing methods can be used to quantify molecules even in the absence of a commercial standard. For these reasons, there is a desperate need to expand the use of NMR in studies of this nature, especially when used alongside cutting‐edge MS‐based tools as this combination represents the ultimate analytical powerhouse for pollutant identification and monitoring.

Seeing as MS is already established as the gold standard for environmental analysis it is clear that the complementary potential of NMR needs promotion. NMR is underutilized in environmental sciences largely due to the lack of available technologies and access, environmental specific expertise/experience, opportunities for hands‐on training and easy to use workflows. The authors would like to make it very clear they are *not recommending NMR be used to replace MS*, but rather that its complementary nature be utilized to help MS in areas where limitations have been identified. For example, in the analysis of fluorinated compounds in firefighting foams, where MS often detects less than 10% of the total ^19^F content present [12]. In these cases, NMR could be used to help identify and quantify components and, in turn, explain why MS struggles (i.e., polymeric form, lack of ionization, and bound unextractable species), while NMR's ability to study samples in situ without concentration or extraction (when required) offers the possibility to explore the impact of sample treatment, which is required for many other analytical approaches, and assist in method optimization.

This part case study and part guide represents a first step toward investing in the use of NMR as a discovery tool in environmental research and to educate readers on some NMR approaches available for these purposes. This proof‐of‐concept study uses NMR to investigate effluents from 38 different industries across the Province of Ontario, Canada. Samples are screened both unconcentrated and after lyophilization. Wastewater effluents from various industrial sectors were analyzed using several 1D and 2D ^1^H/^13^C NMR experiments as well as ^19^F NMR. Finally, low‐field (benchtop) NMR spectroscopy [13] was explored as an accessible alternative to high‐field analysis, as in select cases, it has the resolution and sensitivity to allow monitoring in an economical and easy to use format.

## 2 Experimental

### 2.1 Sample Collection and Preparation

In the fall of 2021, wastewater effluents were collected from a total of 38 sampling locations across Southwestern Ontario, Canada. Several liters of each effluent were vacuum filtered through 20‐μm cellulose fiber filters and stored in sterile 500 mL PET bottles at 253 K. Blanks were collected by filtering and storing deionized water in the same way. Each of the effluent samples was grouped under one of nine industry categories: containerboard, electronics, electroplating, foam insulation, manufacturing, petrochemical, truck washing, wastewater treatment plants (WWTPs), or other. To protect the industries who voluntarily participated in the program very limited information on the industries was provided to the authors.

Sample preparation was performed as follows. Blanks and “natural abundance” samples were prepared by combining 1 mL of filtered water with 1 mg of sodium azide in sterile scintillation vials. From here, 593 μL of this solution was transferred to a new 5‐mm SampleJet NMR tube along with 7 μL of 200‐μM sodium trimethylsilyl propionate (TSP) in D_2_O. Sodium azide was added to suppress microbial activity in the samples, TSP was used as an internal standard for chemical shift calibration, and D_2_O was added to serve as a lock for high‐field NMR experiments. Concentrated samples were prepared by first lyophilizing 1 L of each effluent. This was done in the original PET bottles to limit the risk of contamination through unnecessary transfers. Approximately 83 mg of dried effluent were combined with 1 mL of the respective unaltered (“natural abundance”) effluent and 1 mg of sodium azide in a glass scintillation vial. Like the blanks and non‐concentrated samples, 593 μL of this solution was transferred to a new 5 mm SampleJet tube for analysis, along with 7 μL of 200 μM TSP in D_2_O.

**Transferring from High‐Field (500 MHz) to Low‐Field (80 MHz) NMR**: To further limit the risk of lab‐contamination, samples were not transferred between NMR tubes. However, 5‐mm SampleJet tubes (required for our high‐field autosampler) are approximately 7.4 cm shorter than the standard 5 mm tubes which the 80‐MHz Fourier benchtop spectrometer requires. Thus, a custom adaptor was milled from acetal using a 5‐axis MiRA6 CNC milling machine. One end of this adapter is inserted into the open end of a Sample Jet tube, and the other end is inserted into a cap, providing the additional length needed to position the sample in the coil region inside the 80‐MHz Fourier benchtop NMR spectrometer.

### 2.2 High‐Field NMR Experiments

High‐field spectra were recorded using a 11.7‐T Bruker Avance III‐HD spectrometer equipped with a triple resonance (^1^H, ^13^C, and ^15^N) cryogenic prodigy TCI probe. The spectrometer was tuned to observe frequencies for ^1^H, ^19^F, and ^13^C at 500.3, 470.73, and 125.8 MHz, respectively. All spectra were acquired at 278 K.

**Concentrated samples**: A range of 1D and 2D NMR experiments were run on all preconcentrated samples for compound identification.

**Water suppressed** ^**1**^ **H NMR**: Two different types of water suppression were used in the 1D ^1^H experiments. The first of these was presaturation utilizing relaxation gradients and echoes (PURGE) [14], which provides excellent baseline properties with a very narrow water suppression bandwidth, ideal if resonances of interest fall close to the water signal. In this case an RF field of 50 Hz (0.1 ppm) was applied during the relaxation delay. Collected were 32,768 time domain points with 256 scans, 8 dummy scans, and time of 13.2 s between scans. The latter was calculated using the longest measurable T_1_ in any of the samples (via inversion recovery), such that fully quantitative conditions of 5 × T_1_ were met for all samples. The second sequence was shaped presaturation W5 water suppression by gradient‐tailored excitation (SPR‐W5‐WATERGATE) which is extremely efficient at suppressing broad and intense water common in natural environmental samples that have not been pretreated or concentrated [15]. This approach was included to allow direct comparison between the concentrated and natural abundance samples, the latter which required (SPR‐W5‐WATERGATE) to suppress the broad and intense water signal. Data were collected identically to PURGE. In addition, specific to SPR‐W5‐WATERGATE 125‐μs binomial delay were used putting the W5 sidebands at ~12 and −2 ppm and a perfect echo between the double echo to refocus J‐modulations [16]. Both PURGE and SPR‐W5‐WATERGATE were processed with an zero filling factor of 2 and apodization via an exponential function corresponding to 0.3 Hz line in the transformed spectrum.

**Diffusion editing**: Additionally, a 1D diffusion‐edited experiment utilizing a stimulated echo and LED sequence along with bipolar gradient pulses, two spoil gradients, and presaturation during the relaxation delay was acquired [17]. Diffusion‐edited experiments were collected with a diffusion time of 200 ms and a 2.5‐ms square shaped diffusion gradients corresponding to ~52 G/cm, 512 scans, 32 dummy scans, and 32,768 time domain points. Data were processed with a zero filling factor of 2 and apodization via an exponential function corresponding to 5 Hz line in the transformed spectrum.

**2D homonuclear NMR**: Both COSY and TOCSY experiments were acquired to provide 2D ^1^H‐^1^H correlation information for structure elucidation and matching. COSY was acquired utilizing presaturation during the relaxation delay as well as a W5‐WATERGATE with a 125 μs binomial delay for solvent suppression, along with gradient pulses for selection, 128 increments, each with 32 scans. Data were processed in magnitude mode using unshifted sine‐squared functions and zero filling factors of 2 in both F1 and F2. A phase‐sensitive TOCSY experiment utilizing zero quantum suppression was used.^18^ W5‐WATERGATE with a 125 μs binomial delay was added for solvent suppression. Data were collected using a homonuclear Hartman‐Hahn transfer (DIPSI2 sequence) of 120 ms for mixing over 128 increments, each with 32 scans [18]. Data were processed in a phase sensitive mode using sine‐squared functions that were phase shifted by ᴨ/2 in both F1 and F2, and zero filling factors of 2. Additionally, a J‐resolved ^1^H‐^1^H (JRES) spectrum was obtained. JRES spectra were used to cross confirm the matching processes, and in many cases understand overlap caused by overlapping multiplets. JRES were collected with 16 scans, 128 increments, 80 Hz spectral width in F1. Data were processed in magnitude mode using unshifted sine‐squared functions and zero filling factors of 2 in both F1 and F2.

**2D** ^**1**^ **H‐** ^**13**^ **C heteronuclear NMR**: Heteronuclear 2D experiments are extremely important for 2D NMR given the increased spectral dispersion. Here two heteronuclear NMR experiments are employed for compound identification. The first of these is a phase‐sensitive, sensitivity‐enhanced ^1^H‐^13^C Heteronuclear Single Quantum Coherence (HSQC) experiment that correlates the proton chemical shift to that of any directly attached carbons. Heteronuclear correlations were obtained via a double inept transfer [19] with Echo/Antiecho‐TPPI gradient selection [20]. All inversions and refocusing on the ^13^C channel were achieved using a matched adiabatic pulse pair (Crp60,0.5,20.1 and Crp60comp.4, respectively). Data were collected using a^1^J H‐C of 145 Hz, 4096 time domain points, 64 scans, and 8 dummy scans. Data were processed in phase sensitive mode using sine‐squared functions phase shifted by ᴨ/2 in both F1 and F2, and zero filling factors of 2.

The second ^1^H‐^13^C 2D experiment used was a phase sensitive Heteronuclear Multiple Bond Correlation (HMBC) experiment [21]. Heteronuclear correlations were obtained via heteronuclear zero and double quantum coherence using phase sensitive Echo/Antiecho gradient selection [20]. Refocusing on the ^13^C channel was achieved using an adiabatic Chirp (Crp60comp.4). Data were collected using a ^2,3^J H‐C of 8 Hz, 4096 time domain points, 64 scans, and 8 dummy scans. Data were processed in phase sensitive mode using sine‐squared functions phase shifted by ᴨ/2 in F1, while F2 is processed in magnitude mode (i.e., xfb followed by xf2m).

^**19**^ **F NMR**: In addition, to the ^1^H and ^13^C experiments, ^19^F NMR was also performed given the importance of fluorinated and perfluorinated chemicals in environmental analysis. For general screening, the samples were analyzed using 186,000 time domain points, 51,200 scans, 1.54 s between scans, and16 dummy scans, resulting in a 22.5‐h‐long experiment. These conditions are not fully quantitative but were necessitated for screening given the very low concentration of ^19^F containing species. As discussed in specific sections within this manuscript, additional fully quantitative experiments (delay between scans 30.54 s, 10240 scans, experiment time 3 days 15 h) were performed on select samples, and are discussed in section “3.6.2 ^19^F Quantitative Analysis.” Furthermore, section on “3.6.3 Detection Limits” considers the use of steady‐state free precession NMR [22, 23] in combination with a relaxation agent (10‐mM gadolinium chloride) to optimize data acquisition, where sub‐ppb detection can be achieved in under 15 min. Conditions were used as reported by Gauthier et al. [24]. Due to the presence of a large probe background, spectra were processed using a complete reduction to amplitude frequency table (CRAFT). For this, a Bayesian approach was used to convert the time‐domain FID to a frequency‐amplitude table.

**Processing of 1D SSFP** ^**19**^ **F NMR spectra using CRAFT**: The use of fluorinated compounds in the construction of the cryo probe resulted in a significant probe background that effectively obscured the much smaller signals from the ^19^F containing species that are present in the samples themselves. To overcome this, a complete reduction to amplitude frequency table (CRAFT) was used. Briefly, this technique utilizes Bayesian analysis, rather than a traditional Fourier Transformation to separate the individual signals from a single FID [25, 26]. A detailed outline of the workflow involved in the CRAFT processing used here can be found in Gauthier et al. [24] and an example shown in Figure S1.

^**31**^ **P NMR**: A triple resonance (^1^H, ^13^C, X) broadband probe at 298 K was used to investigate coupling between ^1^H and ^31^P for the identification compounds producing the unusual NMR signals in electroplating effluents (see Section 3.3). For this, a 1D ^31^P inverse‐gated‐decoupling experiment was acquired with 24,576 time domain points, 2048 scans, 8 dummy scans, and a time of 3.2 s between scans. Experiments were acquired with and without WALTZ‐65 decoupling. Similarly, 1D ^1^H SPR‐W5‐WATERGATE experiments with a 125 μs binomial delay were acquired with and without ^31^P WALTZ‐65 decoupling. These used 32,768 time domain points, 128 scans, 8 dummy scans, and a time of 3.2 s between scans. Data was processed with an zero filling factor of 2 and apodization via an exponential function corresponding to 0.3 Hz (^1^H) and 5.0 Hz (^31^P) line in the transformed spectrum.

^**1**^ **H experiments at natural abundance**: Quantitative 1D ^1^H NMR experiments were acquired on blanks and non‐concentrated samples. Due to the intense water signal, W5‐WATERGATE was used for solvent suppression. All conditions for acquisition and processing were identical to those used for the concentrated samples with the exception that 6000 scans were collected (experiment time of ~23 h) given the lower concentration of analytes at natural abundance.

### 2.3 Low‐Field NMR Experiments

Low‐field spectra were acquired using a gradient equipped ^1^H‐^13^C Bruker Fourier 80 Benchtop (80 MHz) NMR spectrometer. Both the magnet and coil temperature were maintained at 298 K. A 1D ^1^H NMR experiment was run on all preconcentrated samples using SPR‐W5‐WATERGATE with an 800‐μs binomial delay for water suppression and 5‐Hz RF field for presaturation. Additionally, various non‐concentrated samples were examined utilizing a 1D ^1^H experiment with W5‐WATERGATE (800‐μs binomial delay) for solvent suppression and a 13.2 s recycle delay for quantification. This was performed to demonstrate that despite the lower sensitivity available through benchtop spectrometers, they still have the potential for examining complex samples in their natural, unaltered states.

### 2.4 Compound Identification

Spectra were calibrated using TSP as the internal standard. Once calibrated, compound identification was done primarily through pattern matching against the Bruker Biofluid Reference Compound Database (versions 2.0.0 through 2.0.9). This was done using Analysis of MIXtures (Amix, v 3.9.15, Bruker Biospin). Matching was done for each preconcentrated sample using the COSY, HSQC, and HMBC experiments. Identified compounds were grouped under different assignment categories depending on the criteria met. To be considered a reasonable candidate, compounds were required to meet the specifications described in Table S1. Pattern matching was done following a very similar method to that described by Anaraki et al. [27].

### 2.5 Quantification

Following the identification of compounds in the preconcentrated samples, select analytes were quantified using Electronic to Access In‐Vivo Concentration (ERETIC2). ERETIC2 refers specifically to a version of ERETIC that does not require specific hardware for electronic references and is best described in Bruker's ERETIC2 manual [28]. ERETIC is well documented in literature [29] and ERETIC2 has even been previously showcased as a tool for environmental quantification [30]. The approach uses the PUCLON relationship that correlates the absolute intensities of two different spectra, such that if the concentration of one is known precisely, the unknown in the other sample can also be calculated using the following equation [29]

$$C_{\mathrm{UNK}}=kC_{\mathrm{REF}}\frac{A_{\mathrm{UNK}}T_{\mathrm{UNK}}\theta_{90}^{\mathrm{UNK}}n_{\mathrm{REF}}}{A_{\mathrm{REF}}T_{\mathrm{REF}}\theta_{90}^{\mathrm{REF}}n_{\mathrm{UNK}}}$$

where the UNK and REF stand for unknown and reference respectively, C is the concentration, T is the temperature, θ_90_ is the 90° pulse length, n is the number of scans used, and k is a correction factor (accounts for different receiver gains and other technical factors). This equation is valid when the experiments are recorded with the same NMR probe and have been tuned and matched. ERETIC2 only needs a 1D spectrum measured on a sample of known concentration, under “quantitative” conditions: a tuned and matched probe, a calibrated 90° pulse, a relaxation delay equal to at least 5 × T_1_, an acquisition time longer than T_2_, and a sufficient signal‐to‐noise ratio. However, given that all concentrations are related back to an “ERETIC standard” the authors recommend testing the approach on a number of “known unknowns” prepared by other lab members in a blind fashion to ensure the approach gives the expected values. As water suppression was acquired for the studies reported here, the ERETIC standards for ^1^H were run using the identical pulse program and spectrometer settings as the environmental samples. In this study numerous test samples for both ^1^H and ^19^F and were confirmed to give the correct absolute value within < 1% error. ^19^F Quantification is considered in more detail in Section 3.6.2 and more considerations as to ^1^H Quantification are discussed below.

## 3 Results and Discussion

### 3.1 Basic Characterization and NMR Techniques

The unrivaled structure elucidation capabilities of NMR make it a powerful technique in discovery‐based research [31, 32]. This is especially true when it is used to complement more routinely employed MS‐based approaches, which typically require preexisting databases in order to confidently identify unknowns [33, 34]. Despite this, NMR is currently underutilized in investigations into environmental media [35].

**FIGURE 1:**
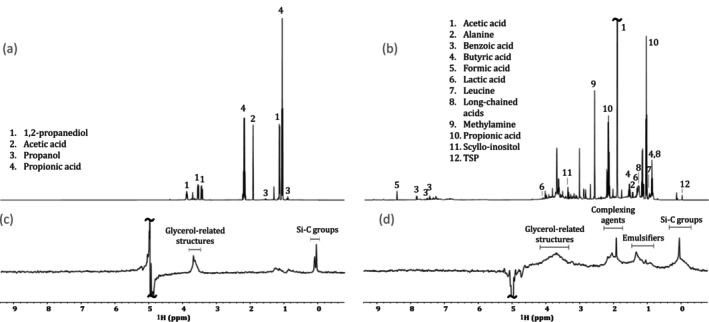
Highlighting the spectral overlap present in samples of varying complexity in standard 1D ^1^H NMR experiments (a,b) and diffusion editing experiments (c,d). In relatively simple samples, such as the effluent from manufacturing—site 1 (a,c), very little overlap is shown, allowing for a straightforward analysis, while more complex samples such as Other—site 4 (b,d), have a much higher degree of overlap and require additional 2D NMR information for characterization.

Structural information available in 1D ^1^H NMR includes chemical shifts, relative integrals, and J‐coupling information [36, 37]. The chemical shift of a specific nucleus describes its chemical environment (reported in units of ppm), whereas J‐coupling (reported in Hz) describes indirect interactions between nuclei [36]. Finally, relative integrals can be used to determine the number of protons represented by a specific signal from the molecule of interest [37]. When examining simple samples with limited spectral overlap, it may be possible to identify and quantify compounds based on the 1D ^1^H spectrum alone. This significantly reduces acquisition times, allowing for a higher throughput. An example of this can be seen in Figure 1a. Here, the industrial effluent from a manufacturing (Manufacturing—site 1) company is dominated by only a few, non‐complex molecules. Thus, compound identification could be done using only the chemical shift information, relative integrals, and J‐coupling constants; all of which are provided by the 1D ^1^H NMR spectrum.

However, most environmentally relevant samples are far more complex, such as the industrial effluent (Other—site 4) shown in Figure 1b. In this case, the number of compounds represented in the 1D ^1^H spectrum is much higher, and the extent of spectral overlap increases dramatically. In cases such as this, reliable identification requires improved spectral dispersion as well as additional information. This can be obtained through the incorporation of supplemental NMR experiments. Some key NMR experiments for compound identification are discussed below, and a summary is included in Table 1. For further discussion see Simpson et al., 2018 [31].

**TABLE 1:** A summary of some key NMR experiments that can be used to identify specific compounds through either database matching or structure elucidation. Example spectra were collected on a real industrial wastewater effluent from Manufacturing—site 1, but only signals from propionic acid are highlighted to make it simpler for readers to follow.

Experiment	Information	Description	Experiment	Information	Description
Diffusion editing 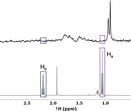	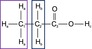 Relatively small molecules, like propionic acid, are filtered out of diffusion edited experiments.	A diffusion edited experiment exploits molecular diffusion to suppress molecules that are free to diffuse within a sample while retaining those with restricted diffusion. This experiment can be used to reduce overlap or identify macromolecules, aggregates or bound species.	Total correlation spectroscopy* 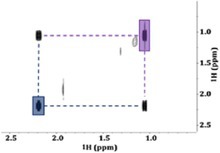 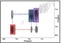	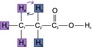 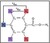 Long‐range correlations*	This experiment utilizes an isotropic mixing period to transfer magnetization between coupled spins. A 2D ^1^H‐^1^H TOCSY experiment detects proton correlations within a molecule or spin system. With an appropriate mixing time, full spin systems can be isolated. This experiment is very useful for structure elucidation and can be helpful for database matching.
Heteronuclear single quantum coherence 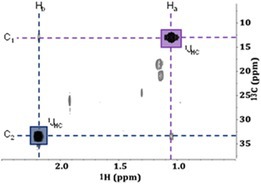	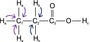 Correlations between protons and carbons that are directly bonded.	^1^H‐^13^C HSQC experiments utilize two INEPT blocks. The first one resulting in antiphase coherence, and the second transforming this into an observable magnetization. The resulting spectra show short range correlations between ^1^H and ^13^C atoms that are directly bonded to each other. This experiment is very useful for structure elucidation and database matching. The additional spectral dispersion from carbon helps overcome spectral overlap.	Heteronuclear multiple bond correlation 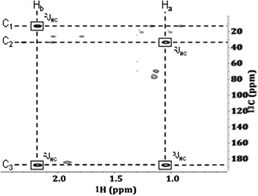	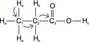 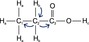	This experiment selects the relatively small long‐range heteronuclear couplings and filters out the much larger single bond couplings. HMBC experiments provide long‐range (2–3 bond) ^1^H‐^13^C through bond interactions. Unlike the HSQC, quaternary carbons are visible in this experiment. This experiment is very useful for structure elucidation and database matching.
Correlation spectroscopy 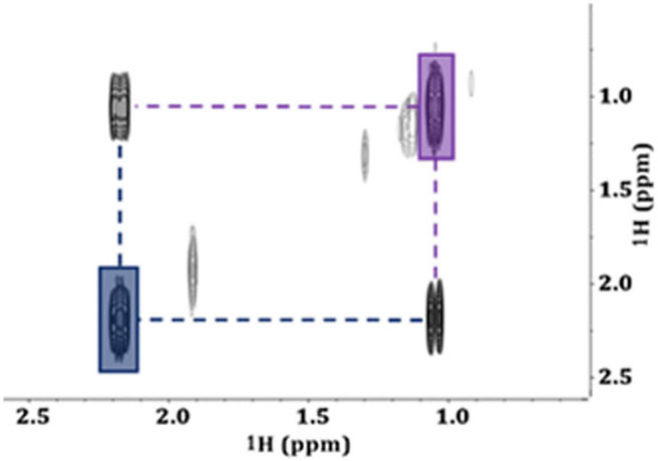	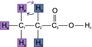 Coherence transferred between protons that are separated by three bonds.	A ^1^H‐^1^H COSY experiment works by transferring coherence between coupled protons. The data generated in this way highlights neighboring protons. This experiment is very useful for structure elucidation and database matching.	J‐resolved 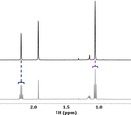	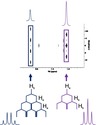	JRES spectra are recorded as 2D experiments, using spin‐echoes and an incremented delay, to separate chemical shift and *J*‐coupling information in separate dimensions. The tilted horizontal projection of this 2D data gives rise to a pure shift spectrum. This experiment is great for reducing overlap.

*Note:* TOCSY and COSY spectra highlighted here show the same information since there are no long‐range ^1^H‐^1^H correlations in propionic acid. A separate example is shown in the gray boxes, where the long‐range ^1^H‐^1^H correlations in benzoic acid can be observed. This spectrum was collected on the effluent from a separate industrial site (Other—site 1).

**Diffusion editing**: Slow‐diffusing molecules can be emphasized through the application of a diffusion‐edited experiment. To do this, the position of all molecules is encoded at the start of the experiment. Following a diffusion period these positions are decoded such that molecules that move rapidly, or diffused through the sample will not be recovered [38, 39]. Thus, the resulting spectrum emphasizes the slow‐moving compounds present in a sample. These slow‐moving compounds are typically large molecules or aggregates [40], both of which result in broad line shapes [41]. In both examples shown in Figure 1, the slow‐diffusing compounds responsible for these broad resonances have a relatively low abundance relative to the faster diffusing components, which are seen clearly in the conventional 1D ^1^H spectra. This indicates that these mixtures are comprised predominately of small molecules rather than larger molecules such as polymers or enzymes. In these particular samples, due to the weak signals from the larger components, identification is challenging, though some tentative possibilities are labeled in Figure 1. However, in cases where larger molecules such as enzymes, polymers, glues (all common in some manufacturing processes) are present in notable concentrations, diffusion editing could be an excellent tool to quickly assess their presence.

**Homonuclear 2D NMR**: Another frequently used method for compound identification is the application of 2D homonuclear and heteronuclear NMR experiments. One of the simplest 2D experiments is ^1^H‐^1^H Correlation Spectroscopy (COSY). Like all 2D experiments, this technique improves spectral dispersion through the introduction of a second dimension. The diagonal of the COSY provides the same information as the conventional 1D ^1^H spectrum, while the cross peaks on either side of this diagonal indicate ^1^H‐^1^H interactions between neighbors [42]. This can be useful for identifying neighboring protons; an important step in the process of structure elucidation. Total Correlation Spectroscopy (TOCSY) similarly provides a 2D spectrum with the 1D information stored along the diagonal. However, with the introduction of a mixing period, long‐range interactions are visible on either side of the diagonal. Thus, entire spin systems can be identified, serving as a relatively simple method of identifying all signals that arise from a molecule (or spin system) [36]. As COSY detects only neighboring protons, the resulting spectrum is very similar irrespective of spectrometer setup making it also ideal for database matching. Conversely, the number of correlations in TOCSY depends on the user selected mixing times (and sample specific conditions such as relaxation). As such TOCSY is complementary and useful for manual confirmation of databases, or key for manually assigning components that are not present in the databases (overlap and sensitivity permitting) given its rich information content covering entire spin systems.

**Heteronuclear 2D NMR**: Despite the improved dispersion brought about by the second ^1^H dimension in both COSY and TOCSY experiments, the relatively narrow chemical shift range accessible by ^1^H NMR (~10 ppm) [36, 43], means that spectral overlap can still be problematic in highly complex samples. However, ^13^C NMR has a chemical shift window of ~200 ppm [43, 44]. As a result, 2D ^1^H‐^13^C experiments provide far greater spectral dispersion. For example, Hertkorn reports the peak capacity 1D ^1^H NMR to be ~3000, whereas ^1^H‐^13^C HSQC can reach ~2,000,000 [45]. Heteronuclear Single Quantum Coherence (HSQC) spectroscopy probes the short‐range interactions between ^1^H and ^13^C nuclei. Thus, the resulting spectrum shows the short‐range interactions between ^1^H nuclei and the ^13^C nuclei to which they are directly bound [31]. Similar to ^1^H‐^1^H COSY it produces results that are largely independent of spectrometer parameters making it ideal for database matching. Long‐range interactions between ^1^H and ^13^C nuclei can be obtained through the application of heteronuclear multiple bond correlation (HMBC) spectroscopy. HMBC correlates proton chemical shifts to carbons that are 2–3 bonds away (including quaternary carbons) and are critical for helping confirm database assignments and mapping out the connectivity of the carbon backbone of a molecule during manual assignment [31].

**J‐resolved NMR**: An additional method that can be used to reduce spectral overlap is a J‐resolved (JRES) experiment. A JRES spectrum is acquired as a 2D experiment where the chemical shift information is stored along the horizontal axis, and the coupling information is preserved in the second dimension [46, 47]. A tilted projection of the resulting spectra can give rise to a ^1^H spectrum devoid of coupling which can be useful for reducing spectral overlap from superimposed multiplets [46].

By combining the aforementioned experiments, it is possible to solve for chemical structures, even in cases of more complex samples. A simple example of the processes involved in manual structure elucidation is shown in Figure S2. This unique capability of NMR makes it highly beneficial in discovery‐based investigations of environmentally relevant media, such as the industrial wastewater effluents investigated here. However, even in relatively simple samples, manual identification is immensely time and labor‐intensive. In the case of very complex mixtures, this is much more challenging and may require additional separations, or the incorporation of higher order (3D–4D) NMR experiments. Such approaches have been proven successful in the analysis of some of the most complex environmental samples. For example, using a combination of 2D hydrophilic interaction chromatography (HILIC) with 2D and 3D NMR experiments, highly oxidized sterols were identified as a major component of dissolved organic matter (DOM) [48]. However, when available, databases offer a quick alternative where previously identified molecules may be of interest.

Database matching provides an effective method for accelerating the characterization of environmentally relevant samples like industrial wastewater effluents. This compound identification strategy has traditionally been used with MS‐based methods of analysis. However, the same approach can be applied to NMR by using chemical shifts and coupling information to identify specific molecules. For the most irrefutable assignments, database matching by NMR is done utilizing a series of 2D experiments. The primary experiments used for database matching (Figures S3 and S4) are the ^1^H‐^1^H COSY, ^1^H‐^13^C HSQC, and ^1^H‐^13^C HMBC, though this list is not exhaustive. Compound identification was done following a rigorous procedure developed by Anaraki et al. [27].

**FIGURE 2:**
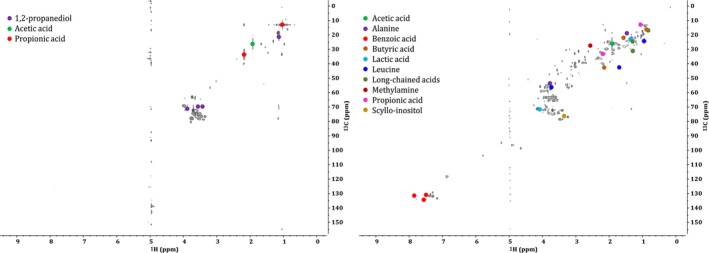
Two example ^1^H‐^13^C HSQC spectra from samples of varying complexity to highlight the compounds that could be identified when matching against a biofluid reference database. In the simple sample from Manufacturing—site 1 (left) many of the most abundant compounds were identified in this way. In the case of the more complex sample from Other—site 4 (right), a wide range of compounds were left unidentified, highlighting the need for further development of environmentally relevant NMR databases.

Figure 2 shows two HSQCs where chemical components were identified using this database‐matching approach. An example of a relatively simple spectrum is shown on the left of this figure. In this example, a large portion of the most abundant compounds was identifiable with available databases. The spectrum on the right represents an industrial effluent with a more complicated chemical makeup. In this case, the majority of contours visible in the spectrum could not be attributed to a specific analyte following the matching procedure. While this kind of database matching shows great potential, current NMR databases are geared toward human metabolites. Thus, further development of environmental specific databases is required for future investigations into environmentally relevant samples. As such, a substantial portion of the compounds present in the industrial wastewater effluent samples could not be identified in this way.

Despite the requirement for further developed databases, when current databases are used alongside manual structure elucidation the two approaches provide a feasible workflow for identifying the components of complex mixtures like industrial wastewater effluents. With increased users and applications of NMR in environmental research, it would be feasible to generate global open‐access databases via collaborative networks which, in turn, would facilitate further use of NMR as a discovery‐based tool [49].

**Quantification using** ^**1**^ **H NMR**: For this study the ^1^H NMR quantification was performed on the unconcentrated samples to demonstrate the feasibility of such analysis. Results are discussed later in specific parts of the manuscript and are included in Table S2. The advantage of collecting data on unconcentrated samples being that the sample is unchanged from its natural state, and any changes from concentration (e.g., loss of volatiles) on drying are avoided. In many ways, the lack of sample preparation required for NMR is one of its great strengths. However, the significant disadvantage is that, given the lower concentration of analytes, analysis at natural abundance demands a much longer analysis time (22 h per sample in this study for natural abundance ^1^H NMR). Further, given that 38 different effluents were studied here, running them in triplicate without concentration was not feasible given that this would have taken an extra 2.5 months of dedicated NMR time just for the ^1^H analysis. As such, while we are confident that the analytical error is less than 1% given blind tests on standards (see methods), the environmental and biological variability in the samples cannot be accounted for in this study given that they could not be run in triplicate. Later in this study ^19^F quantification is also considered (Section 3.6.2), in this case using standard NMR (i.e., no relaxation agents) the concentrations of species were so low that even after concentration the T_1_'s could not be measured. Therefore, attempts at quantification had to use an overly conservative relaxation delay of 30 s leading to 3.5 days of analysis time on each of the examples. On the flipside, as a proof of principle, when optimal relaxation agents were added to a spiked sample and the analysis combined with steady‐state free precession NMR (which optimizes signal per unit time), sub‐ppb detection limits were possible in under 15 min (see Section 3.6.2). The take home message being that it is often a trade off with NMR between keeping the sample intact versus optimizing the analysis time for high throughput. The authors would argue here that the unique abilities of NMR for quantification (a) no‐need to add internal standards (i.e., ERETIC2), (b) every spin (i.e., ^1^H or ^19^F) gives an equal response (i.e., no need for isotopically enriched standards which are often needed by MS), and (c) and the ability to run samples as is (i.e., not concentrated if absolutely essential) provides significant potential for environmental analysis. However, these capabilities are most sensibly employed as a complementary analytical tool.

### 3.2 Effluent Types and Characterization

**FIGURE 3:**
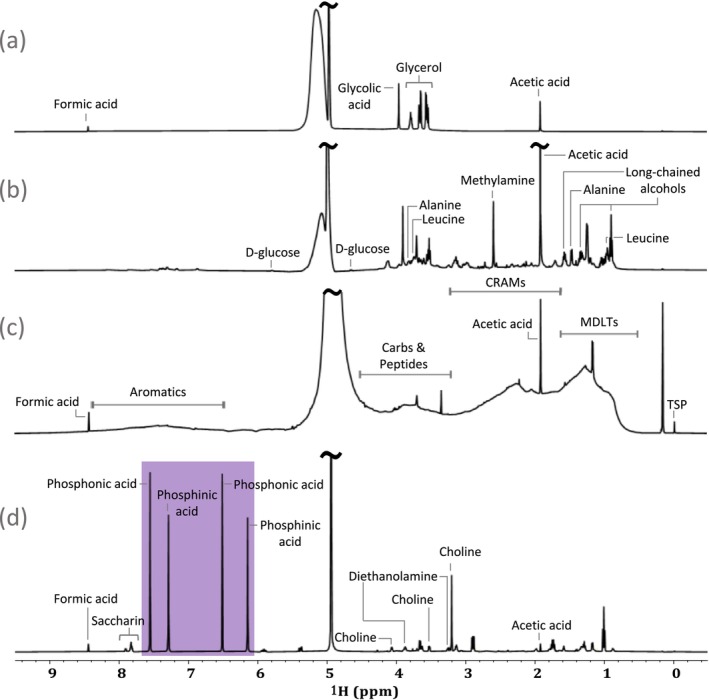
Exploring the varying complexity and resulting spectral profiles of environmentally relevant samples. Electroplating—site 1 is used as an example of the simplest spectral profile (a), Other—site 8 shows a more typical environmental sample with increased complexity (b), and Other—site 9 provides an example of a DOM‐like spectral profile (c), which represents the most complex samples. An example of the final type of spectral profile is shown in (d), this sample is from Electroplating—site 5 and is characterized by the presence of very unique NMR signals which are highlighted in purple.

Like many other environmental samples, the composition of industrial wastewater effluents can vary significantly. In this study the authors found the samples fall into four categories: (a) relatively simple, (b) medium complexity, (c) ultra‐complex, and then (d) samples containing signals of specific interest. Examples of different spectral profiles that may arise from the analysis of industrial effluents are shown in Figure 3. The spectrum shown in (a) is from an electroplating effluent (Electroplating—site 1). This sample represents a simple spectral profile consistent with the sample being dominated by only a few abundant compounds which give rise to minimal spectral overlap. This type of spectral profile is somewhat uncommon in environmentally relevant samples and is fairly simple to interpret.

Figure 3b (Other—site 8) shows a spectral profile that is more typical of environmental samples. In cases such as this, the NMR spectrum is characterized by an increase in complexity due to contributions from a wide range of compounds. As is common in many industrial locations, in this case the effluents are mixed with domestic waste prior to discharge into the environment. As a result, spectral overlap becomes increasingly problematic and signals from the industrial processes are mixed in with biological waste complicating the analysis.

An example of the third type of spectral profile that may be observed in environmental samples is shown in Figure 3c. This effluent is from stormwater (Other—site 9) and displayed a DOM‐like spectral profile, which is characterized by a very high degree of overlap [50, 51]. This could be in part due to the diversity and complexity of inputs as‐well as from reactions [52] and oxidations [50] that can occur in nature.

Spectra with this kind of DOM‐like profile represent the most complex environmental samples. The continuous overlap makes it near impossible to identify specific signals in a 1D spectrum, and often even the improved dispersion of 2D experiments is not enough to separate the signals from individual compounds. As a result, the characterization of these kinds of samples is typically limited to the general categories represented by chemical shift ranges [53]; although detailed NMR analysis of DOM has been possible through the incorporation of hyphenated 2D and 3D NMR approaches [48, 54].

The fourth and final type of spectral profile is defined not by the relative complexity of the sample, but rather by the presence of atypical or unexpected signals. Unique signals, such as those highlighted in Figure 3d (Electroplating—site 5), are indicative of an unexpected or unique pollutant. The presence of atypical signals like these can serve as an indicator that is specific to a particular process, with potential to be used to identify a point source of contamination in the environment.

### 3.3 Spectral Fingerprints “as Clues”

**FIGURE 4:**
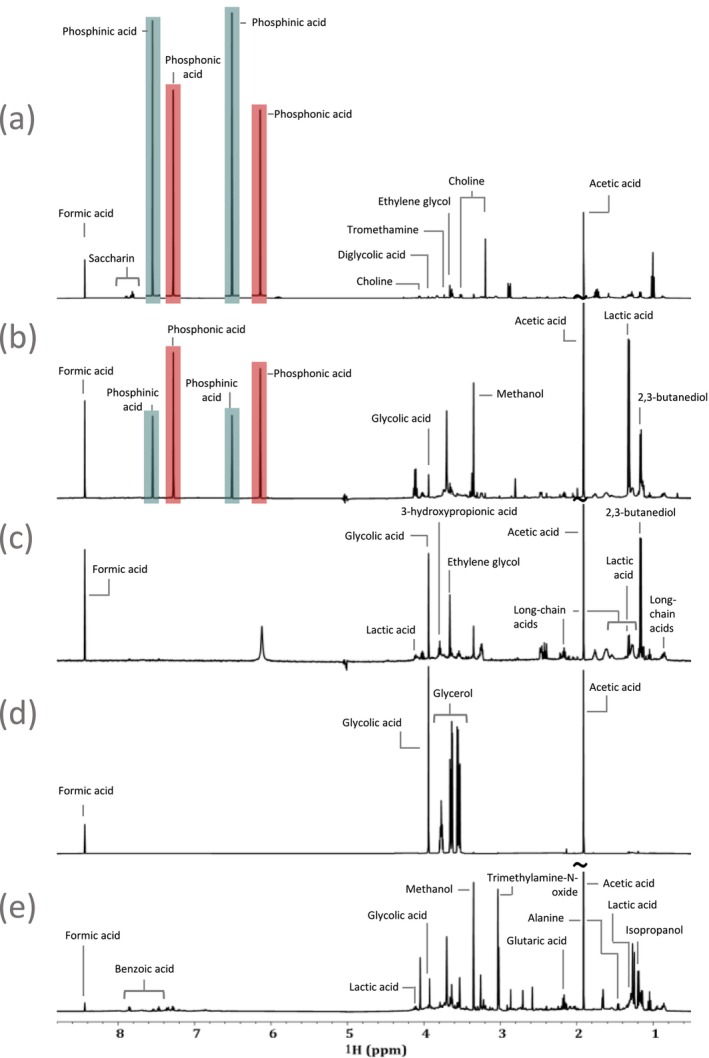
Demonstrating how even within the same overall industrial sector, spectral fingerprints can be used to identify the different processes used at separate sites. The electroplating sector is used as an example to highlight the similarities and differences between the processes used as (a) Electroplating—site 5, (b) Electroplating—site 4, (c) Electroplating—site 3, (d) Electroplating—site 1, and (e) Electroplating—site 2.

Often a key question in environmental research is “where did the pollution come from?” If unique spectral NMR fingerprints exist, they could be used to not just understand the source but also the environmental mixing and reactivity. For example, Figure 3d contains some very interesting and unusual NMR spectral features (see purple highlights), which the authors have never previously seen in environmental samples. This sample is shown again in Figure 4 along with the 1D ^1^H spectra of effluents from several other electroplating industries. Distinct differences can be easily observed in the 1D spectra of these electroplating sites, providing additional insight into the types of processes used at each location. Most interestingly, spectra (a) and (b) have the same unique characteristic signals strongly suggesting these chemicals are directly related to a particular plating process. Given that they are being discharged directly into the environment the identification of these signals becomes an important scientific challenge.

#### 3.3.1 An Example of Discovery

Figure 4a,b share a very distinct signal pattern in the 6.0–8.0 ppm region, suggesting a common electroplating process that is not observed in the remaining sites. These signals did not correspond to any compound present in current NMR databases but were symmetrical between the sets of peaks highlighted in blue and red. This suggests two doublets with J‐couplings of 520 and 570 Hz. However, coupling constants this large are very unusual [55], and are indicative of heteronuclear coupling. This is further supported by the 1D JRES projection and the 2D COSY and TOCSY spectra, which do not provide any indication of ^1^H‐^1^H interactions between these peaks. Additionally, coupling constants as large as these are highly unlikely to be the result of couplings to most of the NMR‐active nuclei that are common to organic compounds, such as ^13^C and ^15^N. This presented an important opportunity to apply a manual approach to compound identification, once again highlighting the power of NMR for discovering the identities of novel and unexpected pollutants.

The signals in blue (Figure 4) are separated by 520 Hz while those in red are separated by 570 Hz in both spectra (a) and (b). As mentioned above, this suggests the presence of two doublets with very large J‐couplings, which appears to be supported by the shared intensity patterns that seem to link the two signals highlighted in red and the two in blue. However, it is also possible that the signals instead arise from four unique singlets.

While there are various ways to confirm J‐couplings the simplest approach is to compare the 1D spectra across different field strengths. When investigating spectra from varying field strengths, there are two things to consider. The first is related to chemical shift. As discussed above, a chemical shift describes the precession frequency (Hz) of a specific nucleus which is impacted by its magnetic environment and thus is field dependent (i.e., it changes based on strength of NMR magnet used) [36]. To account for this field dependency, chemical shifts are described in units of ppm (Hz/MHz), meaning that chemical shifts will remain constant and are characteristic to specific chemical moieties. The second thing to consider is that J‐couplings (Hz) are constant across all field strengths since they are not proportional to the applied magnetic field. As an example, consider a doublet with a J‐coupling of 500 Hz. On a 500‐MHz NMR the two peaks would be 1 ppm part. But the same doublet on an 80‐MHz benchtop system would have peaks 6.25 ppm apart. With this information in mind, if we assume that the four peaks are the result of two doublets, then low‐field data at 80 MHz can be used to confirm this.

**FIGURE 5:**
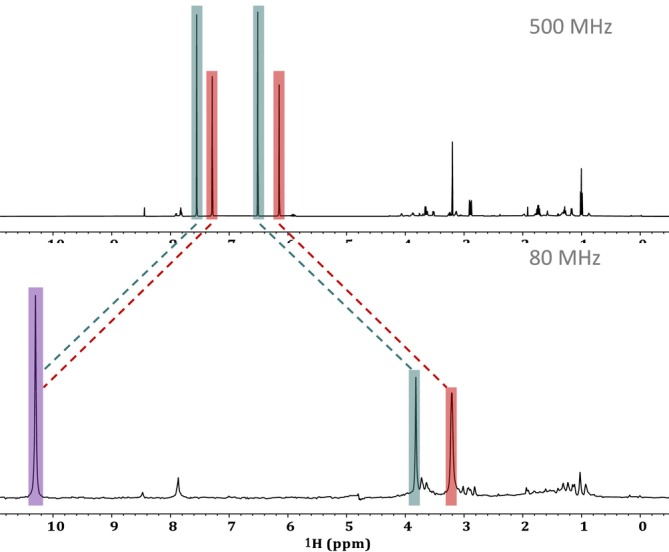
Using a comparison between a high‐field (a) and a low‐field (b) spectrum to determine the multiplicity of the unique signals highlighted in red and blue. Based on the results of low‐field analysis, it could be determined that these signals arise from two separate doublets. The differences in bandwidths (Hz) result in the left‐most peaks of both multiplets overlapping at when analyzed at 80 MHz, this is highlighted in purple.

Figure 5 provides an overlay of a 1D spectra obtained at high field and one at low field. At 500 MHz, the doublet highlighted in red is centered around its chemical shift of ~6.73 ppm. This value will not change with field strength if left in units of ppm, but will be different if reported in Hz. Thus, at 80 MHz, 6.73 ppm is an alias for a chemical shift of 539 Hz. Using the J‐coupling value of 570 Hz, we can deduce that the two peaks of the doublet should appear at ± 285 Hz from this value. This would put the two peaks at 824 Hz (10.3 ppm) and 254 Hz (3.2 ppm). The same logic can be followed for the doublet highlighted in blue, which is centered around 7.05 ppm (564 Hz at 80 MHz) with a J‐coupling constant of 520 Hz. In this case, the two signals should appear at ± 260 Hz from 564 Hz, putting the signals at 824 and 304 Hz (10.3 and 3.8 ppm respectively).

Using this as evidence, the 80‐MHz data (Figure 5) can be easily interpreted. At 80 MHz, the left‐most peaks from both the red and blue doublets fall at 824 Hz (10.3 ppm) giving rise to a single peak with a much larger amplitude. These overlapping peaks are highlighted in purple (Figure 5). The right‐most peaks from the two doublets do not overlap, leaving clear peaks at 3.2 and 3.8 ppm. This provides concrete evidence that the signals arise from two sets of doublets rather than individual singlets.

Despite this progress in the investigation, the structures responsible for these signals remained unknown. None of the standard experiments discussed above provided any clues toward the specific identity of the compounds responsible for the two doublets. However, the standard NMR experiments investigated so far did provide numerous leads that aided in the discovery of the mystery compounds. The first came from the ^1^H‐^1^H TOCSY, which confirmed the presence of two independent compounds as well as the absence of other proton signals in either spin system. The second was from ^1^H‐^13^C HSQC which indicated the protons are not attached to carbons. The third was provided by the ^1^H‐^13^C HMBC, which suggested that no carbon atoms were within 3 bonds of the protons that give rise to these doublets. The fourth clue was in the absence of these signals in the 1D diffusion‐edited experiment. Collectively, these clues indicate the signals of interest likely resulted from two relatively small, inorganic compounds.

From here, the most significant clues available to the investigation were the J‐couplings and chemical shift values. This information, along with insights from the Association of Managers in Magnetic Resonance (AMMRL users group, see acknowledgments) indicated protons directly bonded to a phosphoryl group [56, 57, 58]. To confirm, various 1D ^1^H and ^31^P experiments were acquired both with and without decoupling. When examining the ^1^H spectrum with ^31^P decoupling on (Figure S5), both doublets collapse into singlets, confirming the large coupling constants are the result of ^1^H‐^31^P interactions. A literature review was conducted to identify compounds that are used in electroplating practices and contain this specific functional group. This, along with the ^31^P NMR chemical shifts, and coupling constants identified the compounds as: phosphonic and phosphinic acid [59, 60, 61, 62, 63], Both phosphonic and phosphinic acid are used in copper and nickel electroplating processes, primarily in the production of solar cells. These compounds are added to electroplating baths to be electrolytically reduced in the cell [59, 60]. The ^31^P experiment (Figure S5) revealed two multiplets. The first of these was a doublet with a J‐coupling of 570 Hz. This is consistent with the doublet with a chemical shift of 6.7 ppm in the ^1^H spectra and is indicative of phosphonic acid. The second was a triplet with a J‐coupling of 520 Hz, as was the case for the proton doublet centered around 7.1 ppm, which was confirmed to be phosphinic acid. To our knowledge little is known as to the toxicity or fate of phosphinic and phosphonic acids in the environment. This shows how incorporating evidence from a variety of NMR‐based approaches can allow for the discovery of unknowns. This rather underutilized strength of NMR has the potential to bridge the gap between monitoring known compounds in the environment and discovering the unexpected such as new pollutants and transformation products [64, 65]. Indeed a similar approach was used recently in the discovery of 6PPD‐quinone [6] in the case discussed in section 1. In this case MS provided clues to a problematic chemical being present and NMR was used to search within the mixture and solve the structure of the novel chemical.

#### 3.3.2 Investigating Differences Within the Same Industrial Sector

The signals from phosphonic and phosphinic acid are unique to the two sites shown in spectra (a) and (b) of Figure 4 but were present at different concentrations. In spectra (a) (Electroplating—site 5) phosphonic acid was present with a concentration of approximately 0.666 mM and phosphinic acid at about 0.385 mM, while in (b), Electroplating—site 4, the same compounds were present in at 0.082 mM and 0.024 mM respectively. Despite the similarities these two samples share in regard to these unexpected signals, interesting differences between them point toward contrasting processes and procedures.

For example, (a) (Electroplating—site 5) has notable contributions from saccharin, which is used to improve corrosion resistance when electroplating Ni‐Cr alloys [66], and impacts surface hardness when electroplating with Ni [67, 68]. Saccharin was determined to have a concentration of approximately 0.022 mM. Additionally, signals from choline (0.018 mM) and ethylene glycol (6.599 μM) are also observed in this sample. These two compounds are used together in a solution to influence hardness during Ni deposition onto a brass surface [67, 69]. None of these compounds were detected in (b) (Electroplating—site 4). However, lactic acid, which is used in plating baths for the electroless plating of Ni [70], or to increase Zn deposition during plating processes through complexation [71], was detected in (b) with an approximate concentration of 0.040 mM. The presence of these different compounds strongly suggests that Electroplating—site 4 (b) is involved primarily in electroless plating processes, while Electroplating—site 5 (a) employs a more traditional electroplating process.

The sample represented in Figure 4c (Electroplating –site 3) contains ethylene glycol (2.613 μM), much like the sample represented in (a). However, in the case of (c), choline is not present, suggesting that this site does not employ the same Ni plating process as (a). This is supported by the standard metals analysis provided by the Ontario Ministry of Environment, Conservation, and Parks (MECP) for these two samples. Neither Cr nor Ni were detected in the effluent from Electroplating—site 3. However, in the effluent from Electroplating—site 5, Ni was detected with a concentration of 0.113 mg L^−1^ and Cr at a concentration of 0.247 mg L^−1^. In the absence of choline, ethylene glycol is used as a solvent for non‐aqueous electrodeposition [72]. Additionally, site (c) contained notable contributions from long‐chain (C ≥ 5) acids, which are used in electrocoating processes that protect metals from corroding [73]. These were determined to be present in this sample with a concentration of 2.736 μM. The general description for this site listed electrocoating as one of the primary processes being employed, whereas no other electroplating site fits this description, or contained notable contributions from long‐chain acids.

The sample shown in Figure 4d (Electroplating—site 1) was described as employing processes such as powder coating and screen printing. In this case, the ^1^H spectrum is dominated by glycerol, which was not detected in any other electroplating effluents. Glycerol is used as a plasticizer and is applied prior to a powder coating [74], it was found to have a concentration of 1.118 mM. Additionally, glycerol is often a component of the conductive inks that are used in various printing processes [75].

Finally, Electroplating—site 2 (e) was unique from the other electroplating samples due to the presence of benzoic acid, which was present with a concentration of 0.011 mM. Benzoic acid is typically used to recover chromium from electroplating sludges by separating Fe and Cr through complexation [76, 77]. This is supported by the site description which listed chrome coating as one of the primary operations. Additional details related to the compounds identified in electroplating samples and the concentrations of some such compounds can be found in Table S2.

Ultimately, applying non‐targeted NMR based molecular fingerprinting has the potential to help understand source apportionment, mixing and fate of industrial media in the environment. Such approaches would benefit greatly if databases containing intact environmental mixtures and pure sub‐components were developed over time.

### 3.4 Sample Preparation and Considerations

One of the primary benefits of using NMR for environmental research is its ability to examine complex samples without the need for pretreatment [31]. This eliminates the possibility of unintentionally altering the sample composition during the extraction processes that may be required for other analytical techniques. Additionally, this provides the potential for the real‐time monitoring of effluent compositions or the probing of interactions between different phases [1, 31].

Despite this great benefit, one of the drawbacks of NMR can be its perceived insensitivity compared to techniques like MS. [78] This is less so for 1D NMR of sensitive nuclei such as (^1^H and ^19^F), for example, see section 3.6.2 where sub‐ppb (< 1 ug per L) is demonstrated in under 15 min on a modest 500 MHz NMR using steady state free precession detection. However, for nuclei such as ^13^C, sensitivity is more of a challenge. ^13^C has a gyromagnetic ratio that is approximately one‐fourth that of ^1^H, and a relative abundance of only 1.1% [44, 79]. As a result, ^13^C NMR is far less sensitive than ^1^H NMR. This can become problematic when attempting to identify compounds in a mixture or elucidate the structures of complex molecules if the spectral dispersion provided by ^1^H NMR alone is insufficient. In these cases, 2D experiments such as ^1^H‐^13^C HSQC, and HMBC, can provide the additional spectral dispersion required for structural elucidation, but typically require some form of pretreatment (i.e., lyophilization, SPE etc.). Lyophilization is often considered one the least invasive concentration methods and is commonly used for environmental sample preparation. As ^1^H NMR can be collected before concentration and after concentration it provides an excellent tool to investigate how (or if) the sample preparation changes a sample.

**FIGURE 6:**
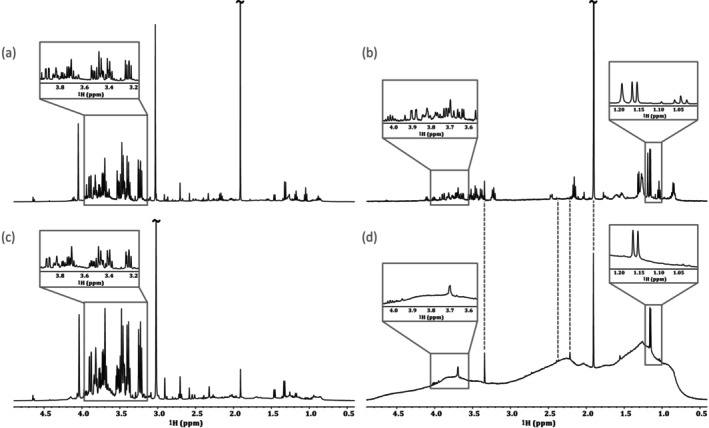
Examining how the NMR spectra of unaltered wastewater effluents (a,b) compared with the NMR spectrum of preconcentrated samples (c,d). In most cases, such as Manufacturing—site 6 (a,c), little no to change is visible in the resulting spectrum aside from an obvious increase in concentration. In rare cases, such as Other—site 9, which was collected from stormwater runoff (b, d) spectral profiles can change dramatically.

In many cases, concentrating a sample through lyophilization does not dramatically alter its spectral profile [15]. For example, the spectrum shown in Figure 6a is the unconcentrated effluent collected from Manufacturing—site 6. Following the lyophilization process, the spectral profile of the concentrated effluent (Figure 6c) is not significantly altered. There are slight changes in intensities of some peaks likely due to the loss of volatiles that can sublime during the freeze‐drying process.

In contrast, there is a dramatic change in the spectral profile of the effluent collected from stormwater discharge (Other—site 9) before and after concentration by lyophilization. At first glance it seems the sample is being completely changed during the freeze‐drying process; however, this is not the case. Closer inspection shows the sharp signals that dominate the spectrum before freeze‐drying are still present, but they are significantly broadened and superimposed on a classic DOM NMR profile. DOM is highly oxidized, highly functionalized organic material with a very high binding capacity and ability to aggregate [80]. On freeze drying one liter of material the DOM is being concentrated from the μg/mL (at natural abundance) to the mg/mL after freeze drying. At this high concentration DOM is known to aggregate and this, along with binding in the mixture, broadened the sharper signal which in turn make the broader DOM profile more prominent in the spectrum. As such, sample preparation requires careful consideration depending on the objective of the study as well as the benefits and limitations of each approach. The approach taken here is to run both natural abundance samples, and concentrated samples, with the natural abundance samples providing a snapshot of the samples “as is” while the concentrated sample provide the mass required for the less sensitive 2D NMR experiments required for confident assignment. Once assigned in the concentrated samples, the same signal can be assigned in 1D NMR at natural abundance. As such this “dual approach” to sample preparation, made possible by the versatility of NMR, provides potential for sample treatment approaches (possibly required by other analytical approaches) to be assessed and optimized.

### 3.5 Other Industry Types

Similarly to the previous discussion of the electroplating effluents, comparisons can be made using the NMR fingerprints of industries that fall under different sectors. In addition to electroplating, 8 separate industrial classes were examined: wastewater treatment plants (WWTPs), containerboard, electronics, manufacturing, commercial truck washing, petrochemical, foam insulation, and “other”. A brief analysis of each of the remaining sectors is provided below. However, additional information and the specific concentrations for some identified compounds can be found in Table S2.

#### 3.5.1 Wastewater Treatment Plants

Samples from two independent WWTPs were included in this study. For each of these sites, a sample of the raw influent and the treated effluent were collected. Figure S6 shows an overlay of the influent and effluent from both sites and includes a bar chart showing the relative changes in concentration following treatment. Similarities were observed between the two sites in terms of the compounds that were identified. However, treatment impacted the relative concentrations of various compounds differently at each site.

Both sites 1 and 2 saw an increase in the concentration of formic acid following treatment. However, while acetic acid increased in site 1 (Untreated influent—1 & Treated effluent—1) from 0.013 mM to 0.016 mM, it decreased in site 2 (Untreated influent—2 & Treated effluent—2) from 0.319 mM to 0.038 mM. Similarly, the concentration of glycolic acid decreased in the treated effluent at site 1 but increased in concentration following treatment at site 2. In both cases, the concentrations of alanine, benzoic acid, and long‐chain acids decreased following the treatment of raw influents.

While it is impossible to discuss the contrasting processes in depth without additional insights into the types of processes applied at the different sites, it should be noted that there are various stages of wastewater treatment, and the practices used at one site may differ significantly from those at another [81, 82]. Thus, it is reasonable to assume that the inconsistencies in the impacts of treatment at sites 1 and 2 likely arise from the differences in the types of treatment employed at each site. For readers interested in more details related to the NMR of WWTPs authors recommend referring to a recent review by Anaraki et al. [79]

#### 3.5.2 Containerboard

The containerboard industry covers the production of both corrugated and solid cardboard. Three samples were collected from separate sites that fall under this category. The 1D ^1^H spectra for these three sites are shown in Figure S7. In all three cases, the majority of identified signals can be attributed to short‐chain (< 5 carbon) acids. This aligns with previous studies, which have documented high concentrations of short‐chain fatty acids in the wastewater derived from containerboard production [83]. All three samples also indicate a large number of signals in the carbohydrate region (3.2–4.5 ppm) [15, 84], likely due to the fiber used to make containerboard [85]. Sites (a) and (b) both contained a notable amount of ethylene glycol, which was not identified in site (c). Additionally, (a) had contributions from tromethamine which is used in the production of paraffin‐based waxes [86] that can be used to coat containerboard [87].

#### 3.5.3 Electronics

Three samples were collected from sites that fell under the industrial sector of electronics. An overlay of the 1D ^1^H NMR spectra from these samples is shown in Figure S8. Sites (b) and (c) shared a relatively similar composition in terms of the compounds identified by database matching. Compounds such as formic acid, dimethylamine, and 2,3‐butanediol were identified in these two sites, but not in (a). This suggests that sites (b) and (c) may employ similar processes and procedures. In (b) (Electronics—site 2), these compounds had concentrations of 6.690 μM, 8.858 μM, and 1.260 μM respectively, while formic acid and dimethylamine were present in concentrations of 0.547 mM and 0.025 mM in the sample shown in spectrum (c) (Electronics—site 3). Based on the presence of 2,3‐butanediol in both sites, it is possible that these sites are involved in the production of conductive silver inks [88]. In contrast, sample (a) appears to have a major contribution of 1‐methyl‐2‐pyrrolidone (4.415 mM), a compound that is sometimes used as a ligand in the production of semiconductor nanoparticles [89].

#### 3.5.4 Manufacturing

Six samples were collected from different manufacturing industries. The resulting 1D spectra (Figure S9) range in complexity. The spectrum shown in Figure S9(a) fits the description of a simple spectrum, as discussed in section 3.3. In this case, the spectrum is dominated by the presence of only a few non‐complex molecules. A similar story can be told for the spectrum shown in (b), which is slightly more complex than (a) but has very little spectral overlap. The remaining spectra (c‐f) increase in complexity and spectral overlap.

Spectrum (d) shows a sample with moderate complexity. In this case, while some overlap is present, specific compounds such as 2,3‐butanediol (3.356 μM), and diglycolic acid can be easily identified, as well as various signals indicative of long‐chain acids (2.989 μM). The most complex of the samples collected from the manufacturing sector appears to be (f). The resulting spectrum contains a great deal of overlap in the carbohydrate region (3.2–4.5 ppm) [15, 84], which has contributions from glucose, as well as various other, unidentified carbohydrates.

Aside from discussions pertaining to the general spectral profiles of each of these samples, an in‐depth explanation or justification for the identified compounds present in each is not possible without more information. Additional insight into the type of products being manufactured (not available to the researchers, to protect the anonymity of the manufacturers who voluntarily participated) at each site would be required to explore the sources of each identified compound.

#### 3.5.5 Industrial Truck Washing

Similar to that observed for the wastewater samples collected from industrial manufacturing sites, samples collected from truck‐washing industries ranged in complexity. Figure S10(a) shows the most complex 1D ^1^H spectrum from a truck washing facility. Here, the carbohydrate‐region and aliphatic region show a great deal of overlap. In the case of (b), the overlap is reduced noticeably, and finally, (c) shows a relatively simple spectrum. In all three cases, signals from long‐chain acids were observed, likely from the application of soaps. Curiously, (c) shows a much lower relative abundance of these long‐chain acids compared to (a) and (b).

Additionally, various amines were identified in all three samples. In both (a) and (b), methylamine and dimethylamine were detected in relatively high concentrations. However, in (c) the tertiary amine, choline was identified as one of the most concentrated components present in the sample. It is possible that this difference is related to the types of detergents used at each site as long‐chain amines are common surfactants [90]. These long‐chain amines can degrade into varying degrees of short‐chain amines. One additional difference worth noting is the presence of terephthalic acid in (a), which had a concentration of 0.075 mM. This acid can be formed by the hydrolysis of waste polyethylene terephthalate [91], which is sometimes used to absorb polycyclic aromatic carbons (PAHs) in the wastewater generated from vehicle washing industries [92]. This suggests the use of some environmental protection processes at site 1 (a).

#### 3.5.6 Petrochemical

Figure S11 shows a comparison between the two wastewater samples collected from petrochemical industries. Both samples have complex 1D ^1^H spectra, however, (b) has a spectral profile approaching the complexity of DOM. Various acids were identified in both samples. For example, lactic acid, which is formed by the microorganisms that are used to degrade n‐alkanes when treating petrochemical wastewater, [93] was identified in both (a) and (b). In spectrum (a), it was present with a concentration of 7.861 μM. In site (b) it was present at 0.012 mM. Ethylene glycol was also found in both samples and has previously been observed in petrochemical wastewater as it is used as an additive in fuel [93, 94]. The sample shown in (a) contains benzoic acid (2.309 uM), which is also sometimes used as a fuel additive [94].

#### 3.5.7 Foam Insulation

Samples from two different foam insulation industries were collected and analyzed. A comparison of the 1D ^1^H NMR spectra from these two sites is shown in Figure S12. In (a), 2,5‐furandicarboxylic acid was detected. This compound is a biomass‐derived alternative to terephthalic acid used in the production of foam insulation in the form of glycol furan dicarboxylate [95]. Urethane was also identified, suggesting the presence of polyurethane foams, which are commonly used as insulation [96].

Various signals from lactic acid, as well as different amino acids, were detected in both samples. It is likely that these originate from greener alternatives to polyurethane or polystyrene foams. For example, alanine and leucine are present in palm kernels [97], rapeseed oil [98], and mustard seeds [99], all of which are used as sources for plant‐based polyols [100]. In addition to these compounds, terephthalic acid, which is used in the manufacturing of polyurethane foams [95, 101], was also identified in (b). Overall, the identified compounds suggest that the effluents from both (a) and (b) may be from industries that are involved in the production of various types of foam insulation.

#### 3.5.8 Other

The final industrial sector included in this study is a general category used to define industries that did not fit under any of the previously discussed industrial sectors. This contained 10 industrial sites ranging from cannabis production to linen cleaning and automotive (Figure S13). Due to the wide range of industries included in this group, a detailed comparison of the similarities and differences between sampling sites is not included. However, a list of the compounds identified in each can be found in Table S2.

### 3.6 ^19^F NMR

Compound discovery is typically done using ^1^H and ^13^C NMR due to their abundance in organic structures [102]. However, in some instances, other NMR‐active nuclei merit additional interest. In terms of environmental research, ^19^F is of high importance given that per‐ and polyfluoroalkyl substances (PFAS)are ubiquitous in soil, water, and air [103]. A myriad of fluorinated chemicals have been introduced into the environment through anthropogenic activities. The use of fluorinated compounds has become widespread across a variety of industrial sectors; such as pharmaceuticals [104], agrochemicals [105], firefighting foams, and many others [106]. As a result, fluorinated compounds have become ubiquitous in the environment and are a frequent topic of discussion. Specifically, PFAS have incurred a great deal of interest due to their persistent and pervasive nature [107, 108, 109].

In terms of NMR, ^19^F is the second most sensitive nucleus after ^1^H and is 100% naturally abundant [79]. Additionally, ^19^F NMR has a chemical shift range of ~700 ppm, resulting in significantly reduced spectral overlap relative to ^1^H NMR. In addition, organo‐fluorine compounds do not naturally occur the environment therefore there is no natural background in ^19^F NMR beyond natural occurring fluoride and some of its salts. This makes ^19^F NMR a relatively simple analysis that is specific to environmentally relevant compounds [110]. For these reasons, the wastewater effluents examined here were also investigated via ^19^F NMR.

#### 3.6.1 ^19^F Qualitative Analysis

As previously described, a triple resonance (^1^H, ^13^C, ^15^N) cryogenic prodigy probe was used to analyze these samples. The proton channel is designed to be tuned to ^19^F, but it is not a dedicated ^19^F probe, and as a result, components used to build the probe (such as Teflon) give rise to a large ^19^F background signal, something not occurring in dedicated ^19^F probes. This background signal overwhelmed the 1D spectrum, effectively burying the smaller, pollutant signals. To overcome this, an alternative processing method to Fourier Transform was employed.

This method, known as complete reduction to amplitude frequency table (CRAFT), utilizes a Bayesian approach to model the decays from individual signals within the FID directly [25]. This allowed the large broad background signals from the probe construction to be filtered out and discarded while still retaining all signals from sharper dissolved species that may be hidden or masked by the probe background (Figure S1). For more information at to the use of this approach for environmental ^19^F NMR please see Gauthier et al. [24]

**FIGURE 7:**
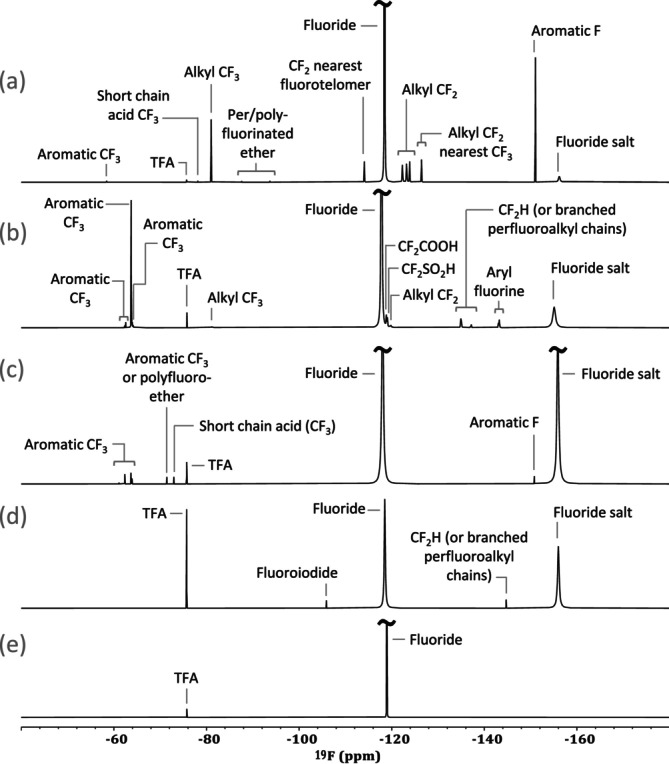
Showing the 1D ^19^F spectra obtained from (a) Electroplating—site 5, (b) Other—site 4, (c) Treated effluent—site 2, (d) Other—site 2, and (e) Manufacturing—site 1 after using CRAFT to processes the raw data.

After CRAFT processing, the data have excellent baselines allowing signals to be easily discriminated (see Figure 7). In some cases, such as the Electroplating sample shown in 7(a), species ranging from simple fluorinated salts to more complex per‐ and polyfluorinated chains can now be observed. In other examples, only a few signals are observed. This is the case for the effluent from a manufacturing industry shown in 7(e).

Identifying the exact structure of the compounds responsible for the observed signals is not trivial [110]. Some can be identified based on the authors experience, and a recently published database developed at the University of Toronto [110]. However, many other compounds can only be described in terms of general characteristics (e.g., aromatic F, fluorinated ether, alkyl CF_3_, etc.)

All wastewater effluent samples were analyzed and the subsequent data processed using this method. Table S3 includes a list of the fluorinated groups identified in each sample with their corresponding chemical shifts. It was found that nearly every sample of industrial effluent analyzed here contained signals from fluoride and trifluoroacetic acid (TFA). The presence of fluoride in these samples in unsurprising given that it is often released through anthropogenic activities and is naturally occurring in high concentrations [111]. The presence of TFA was similarly unsurprising as it is known to be ubiquitous in the environment. Despite the common belief that TFA is a naturally occurring compound, sufficient evidence has not been found to support this claim. However, a range of anthropogenic activities have been shown to result in the formation and release of TFA into the environment, and notably, industrial sites have been highlighted as both primary and secondary sources of TFA. While TFA is thought to have a relatively low toxicity, limited investigations have been done to understand the impact of chronic exposure to low concentration of TFA [112]. The detection of TFA in nearly all samples analyzed here highlights the need for this knowledge gap to be addressed.

**FIGURE 8:**
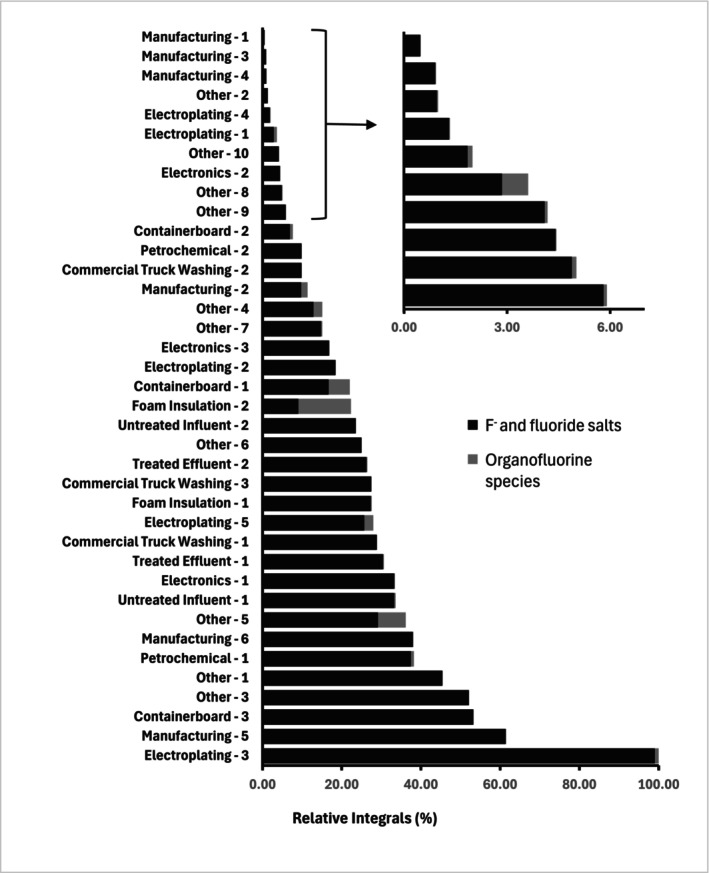
Relative contributions of fluoride and its salts and organofluorine species to the overall absolute integrals of the 1D ^19^F spectra for each of the industrial effluents examined here.

As seen in Figure 8, the industrial effluent with the highest relative concentrations of fluoride and fluoride salts is Electroplating—site 3. This site also contained various signals from both long‐ and short‐chain PFAS.

Of additional interest was the impact of WWTP processes on the distribution of fluorinated species. In sites 1 and 2, signals from additional organofluorine species were present in the treated effluents relative to the raw influents. However, the treatment of Raw Influent—1 resulted in a slight decrease in the relative integral describing all fluorinated species as well as a decrease in the contribution from organofluorine compounds. In contrast, site 2 saw an increase in both the total ^19^F integrals and the overall contribution from organofluorine species with treatment. Previous studies have documented low removal efficiencies or increased concentrations of perfluoroalkyl acids following treatment at municipal WWTPs [113]. This is consistent with the findings for WWTP—site 2, which saw an increase in the number of fluorine signals from short‐chain fluorinated acids specifically.

#### 3.6.2 ^19^F Quantitative Analysis

To show the potential of using a conventional 1D ^19^F experiment for quantitative analysis, two samples were selected for this type of analysis. The samples selected for this analysis were Containerboard—site 1 and Other—site 4. To determine the absolute concentrations of the different signals present in each of these samples, as was previously described for the ^1^H spectra, the electronic reference to access in vivo concentrations (ERETIC2) was used. Table S4 shows the results of this analysis.

However, due to the very low concentrations of fluorinated species present in these samples, and the difficulties associated with the large probe background, this analysis was done using pre‐concentrated samples. Once the concentrations of the specific signals were determined using ERETIC2, a back calculation was done to determine their concentrations in the unaltered effluents. This introduced an additional source of error into the determination of the concentrations in unaltered effluents. The same analysis could theoretically be done using the unaltered samples but would be extremely time consuming and not feasible for routine analysis at least using “standard” NMR approaches. However, the incorporation of more advanced 1D ^19^F experiments could make the quantitative analysis of fluorinated species that are present at low concentrations much more feasible. One such example is explored below.

#### 3.6.3 Detection Limits

Given that ^19^F NMR holds considerable potential for the characterization of unknown PFAS species, limits of detection are well worth considering. In fact, one of the reasons many environmental researchers are hesitant to use NMR for pollutant discovery is its *perceived* lack of sensitivity. The traditional approach to study an unknown sample by NMR, is to try collect quantitative NMR without introducing any additives to a sample. The issue being that as the analytes are at very low concentration, the T_1_ relaxation times cannot be measured. As such, the only option is to use an overly conservative recycle delay (needed to maintain quantitative conditions) which uses spectrometer time very inefficiently and can lead to compromised limits of detection.

**FIGURE 9:**
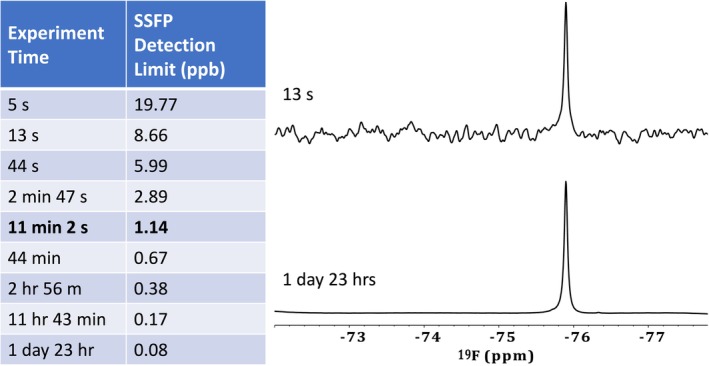
Table showing the detection limits achieved for TFA in the presence of 10‐mM GdCl_3_ using Steady State Free Precession acquisition. Spectra show the spectrum collected in 13 s and 1 day 23 h, which are identical with the exception of the noise, which is averaged over time and is much lower in the longer experiments.

However, it is relatively simple to improve detection limits considerably, by most importantly (a) adding an effective relaxation agent to reduce the T_1_ time and (b) optionally using steady state acquisition [22]. To demonstrate the impact of such a combined approach, we prepared a 1 μM sample of TFA (equivalent to 34 ng in the coil region, ~114 ppb) and added a relaxation agent (10 mM GdCl_3_ [114]). Under these conditions there was enough signal to measure the T_1_ relaxation time which was only 144 ms. Gd is ideal as it greatly reduces T_1_ but has less impact on T_2_, meaning the peaks retain their sharp line shape [115]. As such, a recycle delay of only 720 ms is required to achieve the 5 × T_1_ conditions required for fully quantitative NMR. To further boost the signal, steady‐state conditions can be used. Briefly, spins are subject to a fast train of radiofrequency (RF) pulses spaced by much less than the transverse relaxation time (T_2_). This leads to conditions where the signal never decays to zero and the most intense part of the FID can be acquired continuously. When applied to the TFA sample (see Figure 9), it can be seen that when using SSFP it is *possible to obtain sub‐ppb detection limits within 15 min*; with a detection limit of 80 ppt possible in a 1 day 23 h experiment. Even without SSFP acquisitions, and just the optimal relaxation agent, it was still possible to break the sub‐ppb barrier in ~2 h for TFA using standard fully quantitative NMR. It is also important to note that this is on a relatively old 500 MHz NMR system with a nitrogen‐cryoprobe. With a modern ≥800 MHz system (helium cooled cryo), a factor of 5–10 improvement could be expected. In addition, with state‐of‐the‐art approaches such as CI‐DNP further enhancements of ~500 times have been reported for fluorinated species such as fluorophenol [116]. This suggests that at present, ppt levels are relatively easy to achieve with modest hardware and optimal acquisition, while ppq detection is on the horizon with further development of hyperpolarization methods [117]. Thus, while we would never recommend NMR be used for routine pollutant detection when MS methods are already developed, it is important to document the respectable limits of detection that can be achieved when NMR is needed as a complementary tool [6, 102], and to help dissipate the myth that NMR is too insensitive for environmental contaminant discovery. It is important to note that the values reported here are the instrument detection limits for TFA in the absence of any pre‐concentration procedures.

### 3.7 Low‐Field NMR

The previous sections have explored the potential of using high‐field NMR for the analysis of complex, environmentally relevant samples. Both the identification and quantification of specific chemical pollutants were examined, along with a discussion of the potential of using NMR fingerprints for pollutant tracing. However, this detailed analysis may not always be required. In many instances, process monitoring or simple target screening may be all that is needed. In these cases, it may not be favorable for industries to seek out the expensive and specialized analysis discussed above. Instead, it may be beneficial to explore the potential of low‐field NMR for analyzing industrial effluents, due to their simpler operation and reduced cost [23].

Before delving into the potential of low‐field NMR for the analysis of industrial effluents, there are two main limitations to take into consideration: sensitivity and resolution. The sensitivity of NMR is related to the energy difference between different spin states. Because this energy difference is proportional to the applied magnetic field (B_0_), a magnet with a higher field strength will have higher sensitivity [118]. Thus, low‐field magnets (≤ 100 MHz) [23] will have much lower sensitivity relative to their high‐field counterparts. The second limitation is due to the decreased bandwidth of a low‐field spectrometer as this results in decreased signal dispersion. Consequently, there may be a dramatic increase in spectral overlap at low field. However, with these drawbacks comes with some major benefits, namely, (1) Low‐field benchtop NMR spectrometers use permanent magnets without the need for cryogens, making them (a) economical to purchase and run, (b) accessible in countries where funds and infrastructure for high‐field magnets (e.g., cryogens) may not be available, and (c) compatible with those with metal implants and wheelchairs (field typically contained within the spectrometer housing) (2), are small enough to be potentially placed in the field, with future potential for direct environmental monitoring, (3) commonly include an external lock (separate circuit within the instrument) so liquid samples can be examined as is, (4) are simpler to operate than high‐field systems making them ideal for non‐expert users.

**FIGURE 10:**
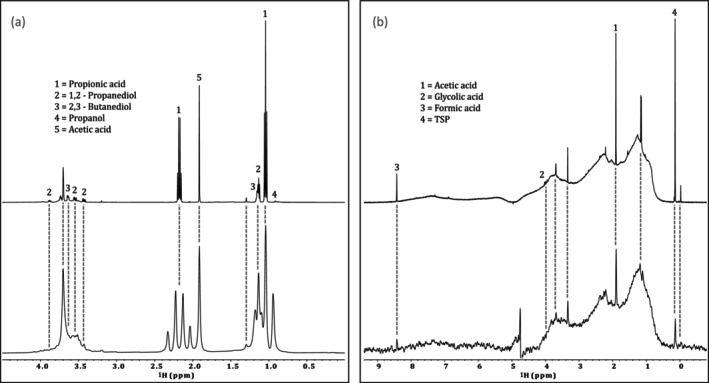
Comparing the high‐field (500 MHz) spectra (top) to low‐field (80 MHz) spectra (bottom) for a simple sample collected from Manufacturing—1 (a) and a sample with a DOM‐like spectral profile (b) collected from Other—9 (storm water).

A comparison of the spectra obtained on the effluent from a Manufacturing plant (Manufacturing—site 1) at 500 and 80 MHz is shown in Figure 10a. In this example, the signals are well resolved at high field, but the reduced bandwidth at 80 MHz results in a broadening of the multiplets. This is because the J‐couplings (Hz) remain constant across different field strengths, as discussed in section 3.3, and the reduced bandwidth means that there is less separation between the signals at 80 MHz. However, even despite this, it clear that once assigned at high field, the lower field spectrum retains enough resolution to allow all of the chemicals to be monitored.

Because of the spectral overlap at low field, a common misconception is that it cannot be used to analyze complex mixtures. However, in the most complex samples (i.e., those with a DOM‐like profile), the reduced spectral overlap at low field does not give rise to any additional barriers. For example, Figure 10b compares the DOM‐like profile of the concentrated effluent collected from Other—site 9 at high and low field. Due to the significant overlap that was observed at high field, the low‐field results are comparable in resolution. Thus, if only broad changes in the spectrum are of interest, a low‐field analysis can be done with comparable success to one at high field. Additionally, if only specific target compounds (or spectral regions) are of interest, previous studies have found great success when applying selective experiments to target compounds in complex samples using low‐field spectrometers [119]. This means that in many cases, low‐field NMR is a promising and affordable alternative for examining industrial effluents, especially if mobile systems are developed in the future. Such systems have potential to screen heavily contaminated media in the field which could then be brought to labs for more traditional MS based profiling.

## 4 Conclusion

When a combination of 1‐ and 2D NMR experiments are used, it is possible to identify a wide range of components in environmentally relevant samples. The unique structure elucidation capabilities of NMR offer the potential for discovering novel and unexpected compounds, such as the phosphoryl acids identified in the two different electroplating effluents, or 6PPD‐quinone in the past [6]. The presence of these specific combinations of experiments that lead to NMR spectral fingerprints could improve the understanding of not just what is in the environment, but how they react and disperse; an important step toward understanding source apportionment and pollutant fate.

Currently, there is an urgent need for environmentally relevant NMR databases to better facilitate large scale studies. Not only would the incorporation of database matching protocols greatly increase throughput, but it could also allow researchers to quickly eliminate all previously identified species thus focusing on the unknown or novel components for manual assignment, a critical step in the discovery of new pollutants and their transformation products.

The results discussed here highlight the potential of using NMR to analyze samples of varying degrees of complexity. For example, sample composition can be compared across different industrial sectors, or, between different processes that occur at sites that fall under the same overarching sector. Similarly, ^19^F NMR was explored for its potential in environmental research for the identification and quantification of fluorinated compounds. Results indicate that ^19^F NMR can provide a relatively simple method of analysis for environmentally relevant issues, with many known and unknown compounds being detected. This is especially valuable for investigations into PFAS, which is a topic of increasing concern. Additionally, using advanced experiments and relaxation agents can give rise to limits of detections that reach the sub‐ppb threshold in less than 15 min at a modest 500‐MHz field. This demonstrates that when fully optimized, NMR sensitivity is sufficient for pollutant discovery, when needed. While NMR can be fully quantitative (without the need for internal standards) the authors would argue these capabilities are best reserved for when cheaper, robust, MS methods are not available or feasible. In such cases NMR could help explain what MS is missing (i.e., lack of ionization), understand association and bindings that may prevent extraction for MS analysis, and target macromolecular species such as polymers of microplastics that can become increasingly challenging to analyze using MS as molecular weight increases [120]. NMR's ability to study samples in situ without concentration or extraction (when required) offers the possibility to explore the impact of sample treatment (required for many other analytical approaches) and assist in method optimization.

Finally, low‐field NMR provides a powerful and accessible alternative to high‐field analysis. Despite the reduced sensitivity at lower field strengths, improved NMR approaches such as CI‐DNP or SSFP can be used to offset these [23, 116]. Consider, for example, if fitted with a flow cell and in combination with ERETIC, a benchtop NMR has the potential to monitor concentrations continuously without internal standards or user intervention. Thus, low‐field NMR analysis opens the potential for real‐time monitoring and the potential for portable spectrometers for in field analysis in the future, or a portable method to identify contaminated media (e.g., total organo‐fluorine) in the field that could then be brought to labs for more traditional MS based profiling. Furthermore, the development of an environmentally relevant low‐field NMR database would greatly benefit the information provided by in‐field analysis.

Ultimately, NMR is an ideal tool for discovery‐based research. Combining a range of experiments can allow for a comprehensive overview of all components of a complex sample and makes the discovery of novel and unexpected compounds possible. Thus, NMR shows great potential for environmental research, especially when used alongside other methods of analysis as a complementary technique.

## Supporting information


**Figure S1.** Visual comparison of (a) the conventional FT ^19^F data to (b) the spectrum reconstructed after CRAFT processing. The NMR Spectra used for this comparison were collected using the wastewater effluent from Foam insulation—site 2. All signals are highlighted in blue and have the corresponding expansions shown on the right. Localized baseline corrections were used for each of the expansions taken from the FT spectrum for ease of comparison.
**Figure S2.** An example of structure elucidation using a combination of 1D and 2D NMR experiments to identify propionic acid. Spectrum (a) provides an overlay of an HSQC (blue) and an HMBC (red) experiment to provide information related to the connectivity of ^1^H and ^13^C nuclei. Spectrum (b) examines the coupling patterns and J‐coupling constants using a 1D ^1^H Spectrum, and (c) shows how a COSY can be used to confirm which protons are within three bonds of each other. When combined the experiments map out the entire H‐C framework of a molecule, thus (resolution and sensitivity permitting) the structures of complete unknowns (including novel molecules) can be mapped out, without any prior knowledge, using these approaches.
**Figure S3.** A visual representation of the matching procedure in AMIX that is used to identify compounds from the bio‐reference databases where (a) shows an example of a list of possible matches when a 2D cross peak of interest is selected. Examples of a good match are shown for an HSQC (b), a 1D ^1^H (c), and a COSY (d) where the spectra in black represent the industrial effluent and those in blue are the spectra included in the biofluid reference databases for benzoic acid. Red arrows in spectra (b) and (d) denote which contour was selected for this match, similar to Anaraki *et al*., 2020.^1^ Note that when performed thoroughly assignments can be made with considerable confidence as HSQC (H‐C correlations), and COSY (H‐H neighbors) provide completely different spectra so a match in both gives an orthogonal and independent confirmation of a structure.
**Figure S4.** Examples of the agreement between chemical shift values of all compounds identified when matching all contours present in a spectrum. To generate these plots chemical shifts from the wastewater effluent spectra are plotted against the chemical shifts obtained from the identified compounds in the biofluid reference databases.^1^ Correlations of 0.99 or greater essentially confirm that the cross peaks from all assigned molecules overlap with the selected peaks in the mixtures. This acts as a filter, indicating any assignments that need to be removed.
**Table S1:** Summary table for confirmed and tentative assignments.
**Figure S5.** Using a combination of (a) 1D ^1^H NMR experiments that are coupled (top) and decoupled (bottom) from ^31^P and (b) 1D ^31^P NMR experiments that are coupled (top) and decoupled (bottom) from ^1^H to confirm the identities of phosphonic and phosphinic acid which are present in the wastewater effluent collected form an electroplating industry (Electroplating—site 5).
**Figure S6.** A comparison of untreated influents (black) and treated effluents (blue) from two separate WWTPs and the changes in the relative integrals of several identified compounds in each. A positive value on the bar chart indicates that the specific compound increased in concentration following wastewater treatment at that plant. Site 1 is shown on the left and site 2 is shown on the right. Urea could only be identified from the 1D chemical shifts (no 2D correlations expected in the experiments performed), therefore the assignment is tentative and colored red to demonstrate this.
**Figure S7.** An overlay of the 1D ^1^H Spectra of the wastewater effluents collected from three separate containerboard industries and some of the compounds identified in each. (a) shows the composition of Containerboard—site 1, (b) the effluent of Containerboard—site 2, and (c) the spectrum from Containerboard—site 3.
**Figure S8.** An overlay of the 1D ^1^H spectra of the wastewater effluents collected from (a) Electronics—site 1, (b) Electronics—site 2, and (c) Electronics—site 3 with some of the identified compounds labeled for each sample.
**Figure S9.** Comparing the compositions of wastewater effluents from (a) Manufacturing—site 1, (b) Manufacturing—site 2, (c) Manufacturing—site 3, (d) Manufacturing—site 4, (e) Manufacturing—site 5, and (f) Manufacturing—site 6.
**Figure S10.** Comparing the effluents collected from (a) Commercial Truck washing—site 1, (b) Commercial Truck washing—site 2, and (c) Commercial Truck washing—site 3.
**Figure S11.** 1D ^1^H NMR spectra of industrial sites (a) Petrochemical—site 1, and (b) Petrochemical—site 2.
**Figure S12.** Using NMR to examine the composition of two unique industrial sites. Spectrum (a) represents Foam Insulation—site 1, and (b) represents Foam Insulation—site 2.
**Figure S13.** Using 1D ^1^H NMR to compare the compositions of wastewater effluents collected from (a) Other—site 1 (Automotive), (b) Other—site 2 (Carbon black), (c) Other—site 3 (Cannabis production), (d) Other—site 4 (Cosmetics), (e) Other—site 5 (Healthcare linen cleaning), (f) Other—site 6 (Personal care products), (g) Other—site 7 (Plastics recycling), (h) Other—site 8 (Refrigeration), (i) Other—site 9 (Industrial storm discharge), and (j) Other—site 10 (Work uniform cleaning).
**Table S2.** Compounds identified in each of the 38 industrial wastewater effluents examined here. Compounds were identified primarily based on database matching and quantification was done on non‐concentrated samples (where possible) using ERETIC2. Not all compounds are quantified due to overlap.
**Table S3.** Characterization of the fluorinated groups detected in the ^19^F NMR experiments following CRAFT processing for each of the 38 industrial effluent samples.
**Table S4.** Example of using ^19^F NMR to quantify the fluorinated species present in two different samples of industrial wastewater effluents. Preconcentrated samples were used for this analysis and the approximate concentrations of each group from the non‐concentrated effluents were calculated based on these results.
**Table S5.** Sample codes and their relationships to previous work.

## Data Availability

The data that support the findings of this study are available from the corresponding author upon reasonable request.
