# Supplementary material for: NMR as a Discovery Tool: Exploration of Industrial Effluents Discharged Into the Environment
Source: Magn Reson Chem. 2025 May 13;63(7):453–75. doi: 10.1002/mrc.5527 (PMC12129648; doi:10.1002/mrc.5527)
Supplement: Supplementary file 1 — Figure S1. Visual comparison of (a) the conventional FT 19F data to (b) the spectrum reconstructed after CRAFT processing. The NMR Spectra used for this comparison were collected using the wastewater effluent from Foam insulation—site 2. All signals are highlighted in blue and have the corresponding expansions shown on the right. Localized baseline corrections were used for each of the expansions taken from the FT spectrum for ease of comparison. Figure S2. An example of structure elucidation using a combination of 1D and 2D NMR experiments to identify propionic acid. Spectrum (a) provides an overlay of an HSQC (blue) and an HMBC (red) experiment to provide information related to the connectivity of 1H and 13C nuclei. Spectrum (b) examines the coupling patterns and J‐coupling constants using a 1D 1H Spectrum, and (c) shows how a COSY can be used to confirm which protons are within three bonds of each other. When combined the experiments map out the entire H‐C framework of a molecule, thus (resolution and sensitivity permitting) the structures of complete unknowns (including novel molecules) can be mapped out, without any prior knowledge, using these approaches. Figure S3. A visual representation of the matching procedure in AMIX that is used to identify compounds from the bio‐reference databases where (a) shows an example of a list of possible matches when a 2D cross peak of interest is selected. Examples of a good match are shown for an HSQC (b), a 1D 1H (c), and a COSY (d) where the spectra in black represent the industrial effluent and those in blue are the spectra included in the biofluid reference databases for benzoic acid. Red arrows in spectra (b) and (d) denote which contour was selected for this match, similar to Anaraki et al., 2020.1 Note that when performed thoroughly assignments can be made with considerable confidence as HSQC (H‐C correlations), and COSY (H‐H neighbors) provide completely different spectra so a match in both gives an orthogonal and in [file MRC-63-453-s001.docx]

NMR as a Discovery Tool: Exploration of Industrial Effluents Discharged into the Environment

Kiera Ronda^1^, Jeremy Gauthier^2^, Khanisha Singaravadivel^1^, Peter M. Costa^1^, Katelyn Downey^1^, William W. Wolff^1^, Daniel H. Lysak^1^, Jacob Pellizari^1^, Owen Vander Meulen,^1^ Katrina Steiner^1^, Amy Jenne^1^, Monica Bastawrous^1^, Zainab Ng^1^, Agnes Haber^3^, Benjamin Goerling^3^, Venita Busse^4^, Falko Busse^4^, Colin Elliot^3^, Scott Mabury^2^, Mohamed Ateia^5^, Derek C. G. Muir^6^, Robert J. Letcher^7^, Krish Krishnamurthy^8^, Sonya Kleywegt^9^, Karl J. Jobst^10^, Myrna J. Simpson^1,2^, and Andre J. Simpson^1,2*^.

^1^Environmental NMR Centre, Department of Physical and Environmental Sciences, University of Toronto Scarborough, Toronto, ON, Canada

^2^Department of Chemistry, University of Toronto, 80 St George Street, Toronto, ON, M5S 3H6, Canada.

^3^Bruker Biospin GmbH, Rudolf-Plank-Str. 23 76275 Ettlingen Germany

^4^Bruker Switzerland AG, Industriestrasse 26,8117 Faellanden, Switzerland.

^5^Department of Chemical and Biomolecular Engineering, Rice University, Houston, TX, 77005, U.S.A.

^6^Canada Centre for Inland Waters, Environment and Climate Change Canada, 867 Lakeshore Rd, Burlington, ON, L7S 1A1, Canada.

^7^Ecotoxicological and Wildlife Health Division, Environment and Climate Change Canada, National Wildlife Research Centre, 1125 Colonel By Drive (Raven Road), Carleton University, Ottawa, ON, K1A 0H3, Canada

^8^Chempacker LLC, 3054 Beckley drive, CA 95135, USA

^9^Technical Assessment and Standards Development Branch, Ontario Ministry of the Environment, Conservation and Parks, Toronto, ON, Canada

^10^Memorial University of Newfoundland, Department of Chemistry, 45 Arctic Ave., St. John’s, NL, A1C 5S7, Canada

*Corresponding Author – [andre.simpson@utoronto.ca](mailto:andre.simpson@utoronto.ca)

Contents

[S1. CRAFT processing 3](#_Toc187939911)

[Figure S1 3](#_Toc187939912)

[S2. Structure elucidation and database matching using NMR Spectroscopy 4](#_Toc187939913)

[S2.1 Manual structure elucidation. 4](#_Toc187939914)

[Figure S2 4](#_Toc187939915)

[Figure S3 5](#_Toc187939916)

[Figure S4 6](#_Toc187939917)

[S2.2 Database matching procedure and confidence levels 6](#_Toc187939918)

[Table S1 6](#_Toc187939919)

[S2.3 Using NMR to determine the identity of unexpected contaminants 7](#_Toc187939920)

[Figure S5 7](#_Toc187939921)

[Figure S6 7](#_Toc187939922)

[S3. Comparisons of NMR spectra from other industries 8](#_Toc187939923)

[Figure S7 8](#_Toc187939924)

[Figure S8 8](#_Toc187939925)

[Figure S9 9](#_Toc187939926)

[Figure S10 10](#_Toc187939927)

[Figure S11 10](#_Toc187939928)

[Figure S12 11](#_Toc187939929)

[Figure S13 11](#_Toc187939930)

[S4. Bringing it all together: a summary of contaminants in wastewater effluents 12](#_Toc187939931)

[S4.1 Protonated species 12](#_Toc187939932)

[Table S2 12](#_Toc187939933)

[S4.2 Fluorinated species 22](#_Toc187939934)

[Table S3 22](#_Toc187939935)

[Table S4 31](#_Toc187939936)

[S5. Sample codes and relationships to previous studies 33](#_Toc187939937)

[Table S5 33](#_Toc187939938)

[References 34](#_Toc187939939)

# S1. CRAFT processing

Figure S1. Visual comparison of (a) the conventional FT ^19^F data to (b) the spectrum reconstructed after CRAFT processing. The NMR Spectra used for this comparison were collected using the wastewater effluent from Foam insulation – site 2. All signals are highlighted in blue and have the corresponding expansions shown on the right. Localized baseline corrections were used for each of the expansions taken from the FT spectrum for ease of comparison.

# S2. Structure elucidation and database matching using NMR Spectroscopy

## S2.1 Manual structure elucidation.

Figure S2. An example of structure elucidation using a combination of 1D and 2D NMR experiments to identify propionic acid. Spectrum (a) provides an overlay of an HSQC (blue) and an HMBC (red) experiment to provide information related to the connectivity of ^1^H and ^13^C nuclei. Spectrum (b) examines the coupling patterns and J-coupling constants using a 1D ^1^H Spectrum, and (c) shows how a COSY can be used to confirm which protons are within three bonds of each other. When combined the experiments map out the entire H-C framework of a molecule, thus (resolution and sensitivity permitting) the structures of complete unknowns (including novel molecules) can be mapped out, without any prior knowledge, using these approaches.

Figure S3. A visual representation of the matching procedure in AMIX that is used to identify compounds from the bio-reference databases where (a) shows an example of a list of possible matches when a 2D cross peak of interest is selected. Examples of a good match are shown for an HSQC (b), a 1D ^1^H (c), and a COSY (d) where the spectra in black represent the industrial effluent and those in blue are the spectra included in the biofluid reference databases for benzoic acid. Red arrows in spectra (b) and (d) denote which contour was selected for this match, similar to Anaraki *et al*., 2020.^1^ Note that when performed thoroughly assignments can be made with considerable confidence as HSQC (H-C correlations), and COSY (H-H neighbors) provide completely different spectra so a match in both gives an orthogonal and independent confirmation of a structure.

Figure S4. Examples of the agreement between chemical shift values of all compounds identified when matching all contours present in a spectrum. To generate these plots chemical shifts from the wastewater effluent spectra are plotted against the chemical shifts obtained from the identified compounds in the biofluid reference databases.^1^ Correlations of 0.99 or greater essentially confirm that the cross peaks from all assigned molecules overlap with the selected peaks in the mixtures. This acts as a filter, indicating any assignments that need to be removed.

## S2.2 Database matching procedure and confidence levels

Database matching was done following the procedure described by Anaraki et al., 2020.^1^ Slight modifications were used in the criteria for classifying the assignments at the different tiers. The criteria used here are outlined in Table S1.

Table S1: Summary table for confirmed and tentative assignments.

| **Scenario** | **Listed in** | **Description** |
| --- | --- | --- |
| Confirmed Assignment | Main Assignment, Table S2 | Requires the observation of key peaks in multiple 2D experiments. These compounds must have previously been documented either as a compound used or produced in the relevant industry. |
| Confident Assignment  Class A | Confident, Table S2, as Class A | Requires the observation of key peaks in multiple 2D experiments, but the compound has not been reported in the literature as being present in industrial effluents or in relevant industrial processes. |
| Confident Assignment  Class B | Confident, Table S2, as Class B | Requires the observation of key peaks in one 2D experiment. These assignments align with 1 or more additional 2D experiments but cannot be made with complete confidence due to spectral overlap. |
| Tentative Assignment | Tentative, Table S2 | Assignments align with multiple NMR experiments but cannot be made with complete confidence due to spectral overlap. |

## S2.3 Using NMR to determine the identity of unexpected contaminants

Figure S5. Using a combination of (a) 1D ^1^H NMR experiments that are coupled (top) and decoupled (bottom) from ^31^P and (b) 1D ^31^P NMR experiments that are coupled (top) and decoupled (bottom) from ^1^H to confirm the identities of phosphonic and phosphinic acid which are present in the wastewater effluent collected form an electroplating industry (Electroplating – site 5).

]

Figure S6. A comparison of untreated influents (black) and treated effluents (blue) from two separate WWTPs and the changes in the relative integrals of several identified compounds in each. A positive value on the bar chart indicates that the specific compound increased in concentration following wastewater treatment at that plant. Site 1 is shown on the left and site 2 is shown on the right. Urea could only be identified from the 1D chemical shifts (no 2D correlations expected in the experiments performed), therefore the assignment is tentative and colored red to demonstrate this.

]

# S3. Comparisons of NMR spectra from other industries

Figure S8. An overlay of the 1D ^1^H spectra of the wastewater effluents collected from (a) Electronics – site 1, (b) Electronics – site 2, and (c) Electronics – site 3 with some of the identified compounds labelled for each sample.

]

Figure S7. An overlay of the 1D ^1^H Spectra of the wastewater effluents collected from three separate containerboard industries and some of the compounds identified in each. (a) shows the composition of Containerboard – site 1, (b) the effluent of Containerboard – site 2, and (c) the spectrum from Containerboard – site 3.

]

Figure S9. Comparing the compositions of wastewater effluents from (a) Manufacturing – site 1, (b) Manufacturing – site 2, (c) Manufacturing – site 3, (d) Manufacturing – site 4, (e) Manufacturing – site 5, and (f) Manufacturing – site 6

]

Figure S10. Comparing the effluents collected from (a) Commercial Truck washing – site 1, (b) Commercial Truck washing – site 2, and (c) Commercial Truck washing – site 3.

]

Figure S11. 1D ^1^H NMR spectra of industrial sites (a) Petrochemical – site 1, and (b) Petrochemical – site 2.

]

Figure S13. Using 1D ^1^H NMR to compare the compositions of wastewater effluents collected from (a) Other – site 1 (Automotive), (b) Other – site 2 (Carbon black), (c) Other – site 3 (Cannabis production), (d) Other – site 4 (Cosmetics), (e) Other – site 5 (Healthcare linen cleaning), (f) Other – site 6 (Personal care products), (g) Other – site 7 (Plastics recycling), (h) Other – site 8 (Refrigeration), (i) Other – site 9 (Industrial storm discharge), and (j) Other – site 10 (Work uniform cleaning).

]

Figure S12. Using NMR to examine the composition of two unique industrial sites. Spectrum (a) represents Foam Insulation – site 1, and (b) represents Foam Insulation – site 2.

]

# S4. Bringing it all together: a summary of contaminants in wastewater effluents

## S4.1 Protonated species

Table S2. Compounds identified in each of the 38 industrial wastewater effluents examined here. Compounds were identified primarily based on database matching and quantification was done on non-concentrated samples (where possible) using ERETIC2. Not all compounds are quantified due to overlap.

| Sample | Confirmed Assignments | Confident Assignments  Class A | Confident Assignments Class B | Tentative Assignments | Spectrum Icon |
| --- | --- | --- | --- | --- | --- |
| Containerboard – site 1 | Acetic acid (0.142 mM)^2^, butyric acid^3,4^, lactic acid (0.037 mM)^5^, long-chain acids (C ≥ 5)^2^, propionic acid (0.031 mM)^2^ | Alanine (5.463 µM), benzoic acid (8.205 µM), beta-hydroxypyruvic acid, diglycolic acid, ethanolamine, ethylene glycol, glycine, trimethylamine-N-oxide | Creatine, D-glucose, dimethylfulfone, hydantoic acid, N-acetylglycine (2.051 µM), thiodiacteic acid, thioglycolic acid, tromethamine | 2-aminobutyric acid, arabitol, betaine, formic acid (0.056 µM), phosphocreatine |  |
| Containerboard – site 2 | 3-hydroxypropionic acid^6^, acetic acid (0.025 mM)^2^, dimethylsulfide^7^, glycolic acid (9.452 µM)^8^ | 2-aminoisobutyric acid, ethylene glycol (1.712 µM), isobarbituric acid | Beta-hydroxypyruvic acid, citric acid, formic acid (0.027 mM), long-chain acids (C ≥ 5) (7.423 µM), terephthalic acid | - |  |
| Containerboard – site 3 | 3-hydroxypropionic acid^6^, acetamide^9^, acetic acid (6.902 µM)^2^, benzoic acid^10^, dimethylamine (1.188 µM)^11^ | 2-hydroxyisobutyric acid, beta-hydroxypyruvic acid, creatine, creatinine, ethylene glycol, glycine, guanidinoacetic acid, methylamine (1.176 µM), N-acetylglycine, phosphocreatine, scyllo-inositol, tromethamine | 1-methyluric acid, alanine, glycolic acid, long-chain acids (C ≥ 5) (0.018 mM), piperazine (1.605 µM), thioglycolic acid | Hydantoic acid |  |
| Electronics – site 1 | 1-methyl-2-pyrrolidone (4.415 mM)^12,13^, acetic acid (0.119 µM)^14^, ethylene glycol^15^ | Creatine, dimethylamine (4.668 µM), ethanolamine, long-chain acids (C ≥ 5), methylamine (0.014 mM), scyllo-inositol | 1,2-propanediol (0.157 mM), 1-penatnol (0.152 mM), acetone, glycine, glycolic acid, guanidinoacetic acid, hydantoic acid, malonic acid, piperazine (0.330 mM), succinic acid, valeric acid | Diethanolamine, methylguanidine, tromethamine |  |
| Electronics – site 2 | Acetic acid (0.014 mM)^14^, diglycolic acid (0.327 µM)^16^, ethylene glycol^15^, formic acid (6.690 µM)^17^ | 1-pentanol, dimethylamine (8.858 µM), scyllo-inositol (0.425 µM) | 2,3-butanediol (1.260 µM), 4-hydroxymandelic acid | Ethanol, phosphocreatine |  |
| Electronics – site 3 | 2,3-butanediol (0.037 mM)^15^, 2-hydroxyisobutyric acid^18^, acetic acid (0.492 mM)^14^, glycine^19^ | 1-pentanol, cystamine, dimethylamine (0.025 mM), guanidinoacetic acid, methylamine (0.010 mM), phosphocreatine, piperazine, sarcosine, scyllo-inositol (0.014 mM), tromethamine | 3-hydroxyisobutyric acid, dimethylsulfone, ethylene glycol, formic acid (0.547 mM), glycolic acid, lactic acid (0.164 mM) | Diglycolic acid, propanol, thiodiacetic acid, thioglycolic acid, trimethylamine-N-oxide (0.014 mM), valeric acid |  |
| Electroplating – site 1 | Acetic acid (0.235 mM)^20^, ethylene glycol^21^, formic acid (0.113 mM)^22^, glycerol (1.118 mM)^23^, glycolic acid^24^, lactic acid (0.011 mM)^25^ | Diglycolic acid | - | - |  |
| Electroplating – site 2 | Acetic acid (0.082 mM)^20^, alanine (9.891 µM)^26^, benzoic acid (0.011 mM)^27,28^, citric acid^29^, creatinine (0.043 mM)^30^, D-mannitol^31^, ethylene glycol^21^, formic acid (8.568 µM)^22^, glutaric acid^32^, glycine^33^, isobutyric acid^34^, isopropanol^35^, lactic acid^25^, methanol^36^, methylamine (9.131 µM)^37^, tartaric acid^38^, thioglycolic acid^39^ | 1-methyluric acid, 2,5-furandicarboxylic acid, 2-hydroxyisobutyric acid, creatine (0.027 mM), diglycolic acid, D-saccharic acid, guanidinoacetic acid, methylguanidine, N-acetylglycine, N-acetyl-L-glutamine, phenylacetic acid, scyllo-inositol, trimethylamine-N-oxide (5.713 µM) | 1,7-dimethyluric acid, 3,7-dimethyluric acid, acetamide, ethylenediamine (0.032 mM), ethylmalonic acid, glycerol, glycolic acid, hydroquinone, isethionic acid, isopropanol, metformin (0.025 mM), piperazine, succinic acid, terephthalic acid, tricine, trimethylamine (1.128 µM), tromethamine | 3-hydroxy-3-methylglutaric acid, 4-aminophenol, 5-aminopentanoic acid, citramalic acid, hippuric acid, leucine, methylacetate, myo-inositol, taurine |  |
| Electroplating – site 3 | Acetic acid (0.012 mM)^20^, benzoic acid^27,28^, D-xylose^40^, ethylene glycol (2.613 µM)^21^, formic acid (0.011 mM)^22^, glycolic acid^24^, isopropanol (5.488 µM)^35^, lactic acid (3.326 µM)^25^, long-chain acids (C ≥ 5) (2.736 µM)^41^, tartaric acid^38^ | 2,3-butanediol (0.879 µM), 3-hydroxypropionic acid, diglycolic acid | 2-aminoisobutyric acid, 3,4-dihydroxybutyric acid, acetone, levulinic acid, scyllo-inositol, succinimide, tromethamine | - |  |
| Electroplating – site 4 | Acetic acid (0.036 mM)^20^, formic acid (0.028 mM)^22^, glycolic acid (5.005 µM)^24^, lactic acid (0.040 mM)^25^, phosphinic acid (0.024 mM)^42^, phosphonic acid (0.082 mM)^42^ | - | 2,3-butanediol, methanol (3.469 µM), tromethamine | - |  |
| Electroplating – site 5 | Acetic acid (0.043 mM)^20^, choline (0.018 mM)^43,44^, dimethylsulfone^45^, ethylene glycol (6.599 µM)^21^, phosphinic acid (0.385 mM)^42^, phosphonic acid (0.666 mM)^42^ | - | Betaine, diglycolic acid, saccharin (0.022 mM) | 2-aminoisobutyric acid, diethanolamine, ethylmalonic acid, formic acid (0.059 mM), isethionic acid, tromethamine (1.976 µM) |  |
| Foam insulation – site 1 | Acetamide^46^, acetic acid (0.142 mM)^46^, acetone^47^, alanine (0.018 mM)^48^, benzoic acid (0.028 mM)^49^, butyric acid^50,51^, dimethylsulfone^52^, ethanolamine^53^, formic acid (0.058 mM)^54^, glycine^55^, lactic acid (0.018 mM)^56^, leucine^48^, piperazine^57^, proline^48,58^, succinic acid (4.109 µM)^59^, tartaric acid^60^, terephthalic acid^61,62^, valine (4.980 µM)^48^ | 2-aminobutyric acid, 2-hydroxyisobutyric acid, 3-hydroxybutyric acid, 3-hydroxyisovaleric acid, 4-deoxyerythronic acid, acetanilide, creatine, creatinine (0.173 mM), diglycolic acid, dimethylamine, D-xylose, ethylmalonic acid, glutamic acid, glycylglycine, guanidinoacetic acid, isethionic acid, L-isoleucine, long-chain acids (C ≥ 5), metformin, methylamine, N-acetylglycine, N-methylhydantoin, propionic acid, sarcosine, scyllo-inositol (2.686 µM), thiodiacetic acid, thioglycolic acid, trimethylamine, trimethylamine-N-oxide, tromethamine, valeric acid | 1-methyluric acid (7.108 µM), 2,3-dihydroxybutyric acid, 2,5-furandicarboxylic acid, 2-aminoisobutyric acid, betaine, cadaverine, ethylenediamine, ethylene glycol, glycolic acid, hydroquinone, isopropanol, methionine, methylguanidine, N-nitrosodimethylamine, N-trimethyllysine, phosphoethanolamine, pyrrolidine, ribitol, succinimide, threitol, tricine, urethane | 1,2,3-butanetriol, choline, D-saccharic acid, D-sorbitol, ethanol, ethyl acetate, glycerol, hexylamine, indoxyl sulfate, mucic acid, pyruvic acid |  |
| Foam insulation – site 2 | Acetic acid (0.489 mM)^46^, alanine (0.076 mM)^48^, benzoic acid (0.014 mM)^49^, formic acid (0.025 mM)^54^, glycine^55^, lactic acid^56^, leucine^48^, piperazine^57^ | Glycolic acid, guanidinoacetic acid, long-chain acids (C ≥ 5), propionic acid (0.143 mM), trimethylamine, | 2-aminoisobutyric acid, betaine, ethylene glycol, ethylmalonic acid, glutamic acid, hydantoic acid, hydroquinone, methylamine (4.146 µM), N-acetylglycine, scyllo-inositol (3.372 µM), terephthalic acid (0.786 µM), thiodiacetic acid, thioglycolic acid, tromethamine | Diglycolic acid |  |
| Manufacturing – site 1 | 1,2-propanediol (5.169 mM)^63^, acetic acid (2.301 mM)^64^, diglycolic acid^65^, formic acid (0.023 mM)^66^, glycolic acid^67^, propionic acid (11.232 mM)^68^, tromethamine^69^ | Scyllo-inositol | 2-aminoisobutyric acid, long-chain acids (C ≥ 5), succinic acid, valeric acid | Ethanol, propanol |  |
| Manufacturing – site 2 | Acetone^70^, ethylene glycol^71^, formic acid (0.041 mM)^66^, tromethamine^69^ | Scyllo-inositol | 2,3-butanediol, isopropanol (1.987 mM) | Acetic acid (0.069 mM), ethanol |  |
| Manufacturing – site 3 | - | - | Acetic acid (0.015 mM), formic acid (5.796 µM), isopropanol (3.261 µM), lactic acid (3.480 µM), methanol (0.958 µM) | Long-chain acids (C ≥ 5) (2.839 µM) |  |
| Manufacturing – site 4 | Acetic acid (0.018 mM)^64^, diglycolic acid^65^, ethylene glycol^71^, formic acid (0.012 mM)^66^, tromethamine^69^ | - | 2,3-butanediol (3.356 µM), 2-aminoisobutyric acid, acetone, long-chain acids (C ≥ 5) (2.989 µM), tartaric acid | Valeric acid |  |
| Manufacturing – site 5 | 1,2-propanediol (1.418 µM)^63^, acetic acid (0.020 mM)^64^, ethylene glycol (0.360 uM)^71^, formic acid (4.364 µM)^66^, tromethamine^69^ | - | Acetamide, lactic acid (2.624 µM), long-chain acids (C ≥ 5) (2.442 µM) | - |  |
| Manufacturing – site 6 | 2-hydroxyisobutyric acid (1.344 µM)^18^, acetic acid (0.072 mM)^64^, alanine (7.005 µM)^72^, benzamide^73^, D-glucose (β: 0.131 mM, α: 0.081 mM)^74^, diglycolic acid^65^, dimethylamine (3.583 µM)^75^, formic acid (0.027 mM)^66^, lactic acid (0.016 mM)^76^, methylamine (3.004 µM)^75^ | Dimethylsulfone, scyllo-inositol | 1-methyluric acid (2.660 µM), 3-hydroxyisovaleric acid, acetamide, acetone, benzoic acid (4.030 µM), glycolic acid, hippuric acid, hydroquinone, thioglycolic acid, trimethylamine | Ethylene glycol, hydantoic acid, N-acetylglycine, piperazine, thiodiacetic acid |  |
| Petrochemical – site 1 | Acetic acid (0.028 mM)^77^, ethylene glycol^77^, formic acid (0.021 mM)^77^, isopropanol^77^ | 3-hydroxypropionic acid (7.401 µM), diglycolic acid, glycolic acid, long-chain acids (C ≥ 5) (5.261 µM) | 1,2-propanediol (4.084 µM), 2-methylserine, acetone, benzoic acid (2.309 µM), lactic acid (7.861 µM), scyllo-inositol (1.414 µM), trimethylamine-N-oxide, | - |  |
| Petrochemical – site 2 | Acetic acid (0.029 mM)^77^, ethylene glycol^77^ | Diglycolic acid, succinimide, tromethamine | 2,3-butanediol (9.977 µM), glycolic acid, succinic acid | Formic acid (0.013 mM), lactic acid (0.012 mM), long-chain acids (C ≥ 5) |  |
| Truck washing – site 1 | Acetic acid (0.811 mM)^78^, ethylene glycol^78^, long-chain acids (C ≥ 5)^78^ | Creatine, dimethylamine, glycolic acid, guanidinoacetic acid, methylamine, N-acetylglycine, propionic acid (0.259 mM), terephthalic acid (0.075 mM), trimethylamine-N-oxide, tromethamine, valeric acid | 1-hexanol, acetamide, betaine, butyric acid, diglycolic acid, hydantoic acid, metformin, phthalic acid, propanol, succinimide, thiodiacetic acid, thioglycolic acid | Alanine |  |
| Truck washing – site 2 | Acetic acid (0.075 mM)^78^, citric acid^79^, ethylene glycol^78^, long-chain acids (C ≥ 5) (0.074 mM)^78^ | 1,7-dimethyluric acid, 1-methylxanthine, 2-hydroxyisobutyric acid, acetamide, alanine, creatine, diglycolic acid, dimethylamine (6.178 µM), ethanolamine, glycine, glycolic acid, leucine, methylamine (0.023 mM), phosphocreatine, piperazine, trimethylamine-N-oxide (2.275 µM), tromethamine | 1-methyluric acid, benzoic acid (0.018 mM), dimethylsulfide (3.257 µM), thiodiacetic acid, thioglycolic acid, tricine | Hydroquinone, formic acid (0.043 mM) |  |
| Truck washing – site 3 | Acetic acid (0.301 mM)^78^, ethylene glycol^78^, long-chain acids (C ≥ 5)^78^ | Choline (0.382 mM), diglycolic acid, formic acid (7.414 µM), glycolic acid, terephthalic acid (0.908 µM), trimethylamine-N-oxide | Acetone (3.351 µM), methanol (0.339 mM), propionic acid (0.020 mM) | - |  |
| WWTP  Raw influent – site 1 | 1,2-propanediol (4.654 µM)^80^, acetic acid (0.013 mM)^81^, alanine (4.018 µM)^82^, aspartic acid^82^, dimethylamine^83^, formic acid (1.924 µM)^81^, glycine^84^, methylamine (0.013 mM)^83^, propionic acid^85^, trimethylamine (0.311 µM)^83^, | Scyllo-inositol | Glycolic acid, leucine, tromethamine | Benzoic acid, ethylene glycol, long-chain acids (C ≥ 5) |  |
| WWTP  Treated effluent – site 1 | 2,3-butanediol^86^, acetamide^87^, acetic acid (0.016 mM)^81^, ethylene glycol^86^, formic acid (7.070 µM)^81^, glycolic acid^88^, methanol (1.856 µM)^85^ | Diglycolic acid | - | Long-chain acids (C ≥ 5) (3.764 µM), propionic acid (2.200 µM) |  |
| WWTP  Raw influent – site 2 | Acetic acid (0.319 mM)^81^, alanine (0.011 mM)^82^, aspartic acid^82^, benzoic acid (3.159 µM)^86^, butyric acid^85^, dimethylamine^83^, ethylene glycol^86^, formic acid (3.587 µM)^81^, glycerol^86^, glycine^84^, glycolic acid^88^, isobutyric acid^89^, leucine^82^, L-isoleucine^87^, long-chain acids (C ≥ 5)^87^, methylamine (0.014 mM)^83^, piperazine^90^, propionic acid (0.045 mM)^85^, sarcosine^82^, terephthalic acid^91^, trimethylamine^83^, tromethamine^92^, valine (4.346 µM)^87^ | Beta-alanine, thioglycolic acid | 5-aminopentanoic acid, dimethylsulfone, ethylmalonic acid, hydroquinone, isethionic acid, scyllo-inositol, thiodiacetic acid, tyrosine, valeric acid | 2-aminoisobutyric acid |  |
| WWTP  Treated effluent – site 2 | 2,3-butanediol (1.949 µM)^86^, 3-hydroxypropionic acid (5.979 µM)^93^, acetamide (0.939 µM)^87^, acetic acid (0.038 mM)^81^, benzoic acid^86^, ethylene glycol^86^, formic acid (0.036 mM)^81^, glycolic acid^88^, lactic acid (6.155 µM)^89^, pyruvic acid^86^ | Diglycolic acid, scyllo-inositol, tromethamine | Levulinic acid, long-chain acids (C ≥ 5) (9.298 µM), methanol | D-mannitol |  |
| Other - site 1  (Automotive) | 2,3-butanediol (9.575 µM)^94^, acetamide^95^, acetic acid (0.068 mM)^96^, benzoic acid (0.022 mM)^97^, dimethylamine^98^, glycerol^99^, piperazine^100^, trimethylamine^101^ | 2,5-furandicarboxylic acid (0.639 µM), alanine (5.904 µM), beta-hydroxypyruvic acid, betaine, creatine, creatinine (0.027 mM), diglycolic acid, D-mannitol, glycine, guanidinoacetic acid, leucine, scyllo-inositol, trimethylamine-N-oxide, tromethamine, valine | 2-hydroxyisobutyric acid, ethylene glycol, galactitol, glycolic acid, isethionic acid, lactic acid (0.010 mM), long-chain acids (C ≥ 5) (0.016 mM), methylamine (4.610 µM) | D-glucose, formic acid (0.049 mM) |  |
| Other - site 2  (Carbon black) | 1,2-propanediol (1.717 µM)^102^, acetic acid (0.012 mM)^103^ | - | 2,3-butanediol | Formic acid (4.983 µM), long-chain acids (C ≥ 5) (2.053 µM) |  |
| Other - site 3  (Cannabis Production) | 2,3-butanediol^104^, alanine^105^, benzoic acid (1.305 µM)^106^, D-glucose^107^, ethylene glycol (1.157 µM)^108^, glycerol^108^, glycine^105^, leucine^105^, succinic acid^109^ | 1-methyluric acid, 2-hydroxyisobutyric acid, acetamide, acetic acid (0.019 mM), creatinine (8.235 µM), diglycolic acid, dimethylamine, ethanolamine, guanidinoacetic acid, methylamine, methylguanidine, N-acetylalanine, piperazine, scyllo-inositol, trimethylamine-N-oxide, beta-hydroxypyruvic acid | Allantoin, betaine, dimethylsulfone, D-mannitol, glycolic acid, hydroquinone, isethionic acid, lactic acid (5.528 µM), methanol, N-acetylglycine, terephthalic acid, tromethamine, | Formic acid (0.010 mM), long-chain acids (C ≥ 5) (0.010 mM) |  |
| Other – site 4  (Cosmetics) | Acetic acid (3.217 mM)^110^, alanine (0.088 mM)^111^, benzoic acid (0.057 mM)^112^, butyric acid (0.287 mM)^113^, creatine^114^, formic acid (0.057 mM)^115^, glycine^111^, guanidinoacetic acid^116^, lactic acid (0.137 mM)^117^, methylamine (0.311 mM)^118^, propionic acid^119^, taurine^120^ | Beta-hydroxypyruvic acid, diglycolic acid, N-acetylglycine, phosphocreatine, piperazine, thiodiacetic acid, trimethylamine-N-oxide | 1-methyluric acid, 2-hydroxyisobutyric acid, betaine, ethylene glycol, glutamic acid, glycolic acid, leucine, metformin (0.097 mM), N-methylhydantoin, scyllo-inositol, terephthalic acid, thioglycolic acid, trimethylamine, tromethamine | - |  |
| Other – site 5  (Healthcare linen cleaning) | 1,2-propanediol (0.375 mM)^121^, acetic acid (5.199 mM)^122^, ethylene glycol^123^, formic acid (0.219 mM)^124^, glycerol^125^, glycolic acid^126^, lactic acid (0.118 mM)^124^, long-chain acids (C ≥ 5)^127^ | D-glucose, diglycolic acid, dimethylsulfone (6.487 µM), terephthalic acid (3.680 µM) | Alanine (0.040 mM), D-sorbitol, ribitol | - |  |
| Other – site 6  (Personal care products) | Acetic acid (0.131 mM)^110^, alanine (0.017 mM)^111^, creatine^114^, dimethylsulfone^128^, ethylene glycol^129^, formic acid (8.002 µM)^129^, glycine^130^, lactic acid^131^, long-chain acids (C ≥ 5) (0.114 mM)^130^ | Piperazine | 2,3-butanediol (0.050 mM), acetone, diglycolic acid, glycolic acid, metformin, thiodiacetic acid, tromethamine | - |  |
| Other – site 7  (Plastics recycling) | 1,3-propanediol^132^, 2,3-butanediol^132^, 3-hydroxyisovaleric acid^133^, acetamide^133^, acetic acid (0.219 mM)^132^, acetone^133^, alanine (0.021 mM)^133^, benzoic acid^134^, butyric acid^135^, diglycolic acid^136^, dimethylamine^137^, ethanolamine^137^, ethylene glycol^138^, glutaric acid^139^, glycine^133^, glycolic acid^133^, hydroquinone^140^, leucine^133^, long-chain acids (C ≥ 5)^139^, methylamine^137^, piperazine^141^, propionic acid (0.077 mMs)^135^, valeric acid^142^ | Isethionic acid, scyllo-inositol, tromethamine | 3-hydroxybutyric acid (0.050 mM), choline, creatine, dimethylsulfone, lactic acid, L-isoleucine, N-methylhydantoin, phosphocreatine, terephthalic acid, thiodiacetic acid, thioglycolic acid, trimethylamine | 2-(4-hydroxyphenyl)propionic acid, betaine, cadaverine, formic acid (8.045 µM), galactitol, isobutyric acid, methylacetate, threitol |  |
| Other – site 8  (refrigeration) | Acetic acid (0.526 mM)^143^, ethanol^143^, ethylene glycol^143^, glycine^144^, long-chain acids (C ≥ 5) ^144^ | 1-hexanol, 1-methyluric acid, 1-pentanol, 2,3-butanediol, 2-aminoisobutyric acid, 2-hydroxyisobutyric acid, 3-hydroxybutyric acid, 3-methylxanthine, alanine, butyric acid, D-glucose, dimethylamine, glycolic acid, leucine, L-isoleucine, methylamine (0.034 mM), phosphocreatine, sarcosine, succinic acid, terephthalic acid, trimethylamine, trimethylamine-N-oxide, tromethamine, valine | Acetone, betaine, creatine, diglycolic acid, dimethylsulfone, heptanol, isobutyric acid, isopropanol, isovaleric acid, N-acetylglycine, N-methylhydantoin, propanol, propionic acid, pyruvic acid, scyllo-inositol (2.814 µM) | 3-hydroxyisovaleric acid, formic acid (0.016 mM) |  |
| Other – site 9  (Industrial storm discharge) | Acetic acid (0.052 mM)^145^ | - | Acetone, diglycolic acid, glycolic acid, tromethamine | 2,3-butanediol, benzoic acid (1.827 µM), D-glucose (β: 6.933 µM, α: 4.435 µM), formic acid (0.034 mM), glycerol, propionic acid (3.584 µM) |  |
| Other – site 10  (Work uniform cleaning) | Acetic acid (0.818 mM)^122^, betaine^146^, ethylene glycol^123^, heptanol^147^, formic acid (0.021 mM)^124^, long-chain acids (C ≥ 5)^127^ | 1-penatnol, 2,3-butanediol, 3-hydroxybutyric acid (0.041 mM), acetamide, diglycolic acid, dimethylamine (2.745 µM), propionic acid (0.088 mM), scyllo-inositol, terephthalic acid, valeric acid | 1,3-propanediol, 1-hexanol, 3-hydroxypropionic acid, ethanol, glutaric acid, glycolic acid, isocaproic acid, propanol | - |  |

## S4.2 Fluorinated species

Table S3. Characterization of the fluorinated groups detected in the ^19^F NMR experiments following CRAFT processing for each of the 38 industrial effluent samples.

| Sample | Chemical shift (ppm) | Assignment | Spectrum Icon |
| --- | --- | --- | --- |
| Containerboard – site 1 | -147.1  -118.8  -92.9  -91.3  -82.7 to -80.7  -75.8  -57.5  -55.8 | Aromatic -F  F^-^  Per/polyfluoro ether  Per/polyfluoro ether  Alkyl -CF_3_  TFA  Aromatic -CF_3_  Aromatic -CF_3_ |  |
| Containerboard – site 2 | -130.2  -118.9  -83.5  -75.7  -58.2 | -CF_2_H  F^-^  Alkyl -CF_3_  TFA  Aromatic -CF_3_ |  |
| Containerboard – site 3 | -155.0  -118.3  -81.1  -75.8  -72.2 | Fluoride salt  F^-^  Alkyl -CF_3_  TFA  Short chain fluorinated acid (-CF_3_) |  |
| Electronics – site 1 | -155.3  -150.5  -118.2  -96.0  -94.5  -78.2  -75.8  -63.8 | Fluoride salt  Aromatic -F  F^-^  Per/polyfluorinated ether  Per/polyfluorinated ether  Short chain acid (-CF_3_)  TFA  Aromatic -CF_3_ |  |
| Electronics – site 2 | -155.4  -118.2  -109.8  -75.8 | Fluoride salt  F^-^  Likely two aromatic rings with fluorine (similar to BPA-AF)  TFA |  |
| Electronics – site 3 | -155.4  -141.7  -137.1  -133.9  -118.3  -82.5  -75.7  -63.7 | Fluoride salt  Aryl fluorine  -CF_2_H  -CF_2_H  F^-^  Alkyl -CF_3_  TFA  Aromatic -CF_3_ |  |
| Electroplating – site 1 | -150.8  -118.8  -75.8 | Aromatic F  F^-^  TFA |  |
| Electroplating – site 2 | -150.4  -118.0  -75.8  -63.8 | Aromatic F  F^-^  TFA  Aromatic -CF_3_ |  |
| Electroplating – site 3 | -155.2  -121.5  -118.3  -83.6  -81.1  -76.3 to -75.9  -75.8  -75.6 | Fluoride salt  Alkyl -CF_2_-  F^-^  Alkyl -CF_3_  Alkyl -CF_3_  Short chain acid (-CF_3_)  TFA  Short chain acid (-CF_3_) |  |
| Electroplating – site 4 | -156.6  -151.1  -140.5  -133.3  -128.8  -118.9  -104.7  -81.0 to -79.7  -75.7  -57.8 | Fluoride salt  Aromatic -F  -CF_2_H  -CF_2_H  Alkyl CF_3_  F^-^  Likely two aromatic rings with fluorine (similar to BPA-AF)  Alkyl -CF_3_  TFA  Aromatic -CF_3_ |  |
| Electroplating – site 5 | -156.2  -151.0  -126.4  -123.9 to -122.3  -118.5  -114.1  -93.7  -87.6  -81.0  -78.1  -75.7  -58.4 | Fluoride salt  Aromatic -F  Alkyl nearest -CF_3_ (well defined triplet)  Alkyl -CF_2_-  F^-^  -CF_2_- near fluorotelomer (well defined triplet)  Per/polyfluorinated ether  Per/polyfluorinated ether  Alkyl -CF_3_  Short chain acid (-CF_3_)  TFA  Aromatic -CF_3_ |  |
| Foam insulation – site 1 | -155.3  -118.4  -114.0  -112.1  -75.8 | Fluoride salt  F^-^  -CF_2_- near fluorotelomer  -CF_2_- near fluorotelomer  TFA |  |
| Foam insulation – site 2 | -155.6  -144.5  -143.7  -137.2 to -135.6  -120.1 to -119.1  -118.3  -106.4 to -101.9  -75.8  -64.2 to -62.3 | Fluoride salt  Aromatic -F  Aromatic -F  -CF_2_H  -CF_2_- near carboxylic or sulfonic acid  F^-^  Likely two aromatic rings with fluorine (similar to BPA-AF)  TFA  Aromatic -CF_3_ |  |
| Manufacturing – site 1 | -118.9  -75.7 | F^-^  TFA |  |
| Manufacturing – site 2 | -155.6  -118.8  -79.1  -75.8 | Fluoride salt  F^-^  Alkyl -CF_3_  TFA |  |
| Manufacturing – site 3 | -156.3  -143.5  -118.9  -111.0  -93.1 to -90.9  -80.9  -75.7  -67.7 | Fluoride salt  Aromatic -F  F^-^  Likely two aromatic rings with fluorine (similar to BPA-AF)  Per/polyfluorinated ether  Alkyl -CF_3_  TFA  Aromatic -CF_3_ |  |
| Manufacturing – site 4 | -118.8  -75.9 | F^-^  TFA |  |
| Manufacturing – site 5 | -155.4  -141.1  -134.0  -133.4  -118.5  -87.6  -75.8 | Fluoride salt  Aromatic -F  -CF_2_H  -CF_2_H  F^-^  Per/polyfluorinated ether  TFA |  |
| Manufacturing – site 6 | -155.2  -117.9  -75.8  -63.9 | Fluoride salt  F^-^  TFA  Aromatic -CF_3_ |  |
| Petrochemical – site 1 | -155.5  -128.1  -121.4  -118.5  -83.5 to -80.9  -76.2  -75.9  -75.8  -73.2 | Fluoride salt  -CF_2_- nearest terminal -CF_3_  Alkyl -CF_2_-  F^-^  Alkyl -CF_3_  Short chain acid (-CF_3_)  Short chain acid (-CF_3_)  TFA  Short chain acid (-CF_3_) |  |
| Petrochemical – site 2 | -155.9  -118.7  -75.7  -59.0 to -55.8 | Fluoride salt  F^-^  TFA  Aromatic -CF_3_ |  |
| Truck washing – site 1 | -156.3  -118.9  -75.8 | Fluoride salt  F^-^  TFA |  |
| Truck washing – site 2 | -152.8  -151.7  -118.6  -75.8  -63.5 | Aromatic -F  Aromatic -F  F^-^  TFA  Aromatic -CF_3_ |  |
| Truck washing – site 3 | -155.9  -118.9  -75.8 | Fluoride salt  F^-^  TFA |  |
| WWTP  Raw influent – site 1 | -155.6  -135.7  -118.5  -83.5  -75.7  -63.7 to -62.4 | Fluoride salt  -CF_2_H  F^-^  Alkyl -CF_3_  TFA  Aromatic -CF_3_ |  |
| WWTP  Treated effluent – site 1 | -155.6  -143.3  -135.1  -117.6  -85.2  -81.4 to -81.2  -75.7  -63.9 to -62.2 | Fluoride salt  Aromatic -F  -CF_2_H  F^-^  Per/polyfluorinated ether  Alkyl -CF_3_  TFA  Aromatic -CF_3_ |  |
| WWTP  Raw influent – site 2 | -155.8  -118.7  -63.7 | Fluoride salt  F^-^  Aromatic -CF_3_ |  |
| WWTP  Treated effluent – site 2 | -155.9  -150.8  -118.0  -75.7  -72.9  -71.4  -63.7 to -61.1 | Fluoride salt  Aromatic -F  F^-^  TFA  Short chain acid (-CF_3_)  Short chain acid (-CF_3_)  Aromatic -CF_3_ |  |
| Other – site 1  (Automotive) | -155.8  -118.5  -75.7 | Fluoride salt  F^-^  TFA |  |
| Other – site 2  (Carbon black) | -156.0  -144.7  -118.5  -105.9  -75.7 | Fluoride salt  -CF_2_H  F^-^  Fluoroiodide (R-CF_2_-I)  TFA |  |
| Other – site 3  (Cannabis Production) | -155.4  -118.3  -78.6  -75.8  -68.9 | Fluoride salt  F^-^  Short chain fluorinated acid (-CF_3_)  TFA  Aromatic -CF_3_ |  |
| Other – site 4  (Cosmetics) | -155.1  -143.2  -143.0  -137.2 to -134.9  -119.8  -119.1  -118.8  -81.2  -75.8  -64.2 to -62.3 | Fluoride salt  Aryl -F  Aryl -F  -CF_2_H  Alkyl -CF_2_-  -CF_2_-SO_2_H  F^-^  Alkyl -CF_3_  TFA  Aromatic -CF_3_ |  |
| Other – site 5  (Healthcare linen cleaning) | -161.6  -160.6  -156.1  -148.4 to -142.6  -132.3  -118.9  -83.5 to -81.3  -76.1  -75.7  -74.1 to -71.4  -63.7 to -57.9 | Aromatic -F  Aromatic -F  Fluoride salt  Aromatic -F  -CF_2_H  F^-^  Alkyl -CF_3_  Short chain acid (-CF_3_)  TFA  Short chain acid (-CF_3_)  Aromatic -CF_3_ |  |
| Other – site 6  (Personal care products) | -155.6  -118.6  -61.6 | Fluoride salt  F^-^  Aromatic -CF_3_ |  |
| Other – site 7  (Plastics recycling) | -155.5  -150.5 to -147.0  -133.8  -118.7  -106.2  -105.1  -99.6 to -96.7  -79.5  -75.8  -72.9  -71.4  -66.5  -61.9 | Fluoride salt  Aromatic -F  -CF_2_H  F^-^  Likely two aromatic rings with fluorine (similar to BPA-AF)  Likely two aromatic rings with fluorine (similar to BPA-AF)  Per/polyfluorinated ether  Alkyl -CF_3_  TFA  Short chain acid (-CF_3_)  Short chain acid (-CF_3_)  Aromatic – CF_3_  Aromatic – CF_3_ |  |
| Other – site 8  (Refrigeration) | -155.4  -139.4  -118.4  -112.1  -84.2  -81.3  -75.8  -64.2 to -57.7 | Fluoride salt  -CF_2_H  F^-^  Alkyl -CF_2_- nearest functional group  Per/polyfluorinated ether  Alkyl -CF_3_  TFA  Aromatic -CF_3_ |  |
| Other – site 9  (Industrial storm discharge) | -155.8  -149.2  -133.9  -118.7  -94.6  -75.8 | Fluoride salt  Aromatic -F  -CF_2_H  F^-^  Per/polyfluorinated ether  TFA |  |
| Other – site 10  (Work uniform cleaning) | -156.7  -151.2  -151.1  -118.9  -94.5  -93.7  -75.6  -73.8 | Fluoride salt  Aromatic -F  Aromatic -F  F^-^  Per/polyfluorinated ether  Per/polyfluorinated ether  TFA  Short chain acid (-CF_3_) |  |

Table S4. Example of using ^19^F NMR to quantify the fluorinated species present in two different samples of industrial wastewater effluents. Preconcentrated samples were used for this analysis and the approximate concentrations of each group from the non-concentrated effluents were calculated based on these results.

| Sample | M_total obtained from 1 L_  (g) | Chemical shift (ppm) | Assignment | M_total used in conc_  (g) | C_conc_  (µM) | Approximate C_non-conc_  (µM) |
| --- | --- | --- | --- | --- | --- | --- |
| Containerboard – site 1 | 0.5706 | -155 | Fluoride salt | 0.0840 | 116.12 | 1.13 |
|  |  | -147 | Aromatic -F |  | 33.09 | 0.22 |
|  |  | -119 | F- |  | 1636 | 11.11 |
|  |  | -84 to -79 | Alkyl -CF_3_ |  | 49.78 | 0.34 |
|  |  | -75 | TFA |  | 0.52 | 3.55$\times{10}^{-3}$ |
|  |  | -61 to -53 | Aromatic -CF_3_ |  | 4.78 | 0.032 |
| Other – site 4  (Cosmetics) | 0.3299 | -155 | Fluoride salt | 0.0824 | 571 | 2.29 |
|  |  | -117 | F- |  | 52.98 | 0.21 |
|  |  | -143 | Arly fluorine |  | 20.04 | 0.080 |
|  |  | -137 to -134 | -CF_2_H- |  | 9.71 | 0.078 |
|  |  | -81 | Alkyl -CF_3_ |  | 2.50 | 0.030 |
|  |  | -76 | TFA |  | 1.62 | 0.019 |
|  |  | -64 to -62 | Aromatic -CF_3_ |  | 15.52 | 0.186 |

Calculating the concentration of fluorinated species present in the non-concentrated effluents based on the quantitative results from the pre-concentrated samples:s

$0.001 L\times C_{conc}=n_{conc}$ (1)

$C_{conc}=concentration in 1 mL of concentrated sample$

$n_{conc}=number of moles in 1 mL of concentrated sample$

$\frac{n_{conc}}{m_{total used in conc}}=Y \frac{mol}{g}$ (2)

$m_{total used in conc}=total dry mass used to make 1 mL of concetrated$

$Y=ratio of moles of compound per dry mass$

$m_{total obtained from 1 L}\times Y \frac{mol}{g}= n_{non-conc}$ (3)

$n_{non-conc}=number of moles of compound in total mass obtained from lyophilizing 1 L of wastewater$

$\frac{n_{non-conc}}{1 L}=C_{non-conc}$ (4)

$C_{conc}=Concentration in the unaltered wastewater (before lyophilization)$

# S5. Sample codes and relationships to previous studies

Table S5. Sample codes and their relationships to previous work.

| **Industry code** | **Industry** | **As used in previous work** |
| --- | --- | --- |
| Containerboard - site 1 | Containerboard | B9^148^, Containerboard 1 ^149^ |
| Containerboard - site 2 | Containerboard | B2^148^, Containerboard 2 ^149^ |
| Containerboard - site 3 | Containerboard | B1^148^, Containerboard 3 ^149^ |
| Electronics - site 1 | Electronics | A9^150^, Circuit boards 1 ^149^ |
| Electronics - site 2 | Electronics | A10^150^, Circuit boards 2 ^149^ |
| Electronics - site 3 | Electronics | Computers/semiconductors ^149^ |
| Electroplating - site 1 | Electroplating | B6^148^, Electroplating 1 ^149^ |
| Electroplating - site 2 | Electroplating | Electroplating 2 ^149^ |
| Electronics - site 3 | Electronics | Computers/semiconductors ^149^ |
| Electroplating - site 4 | Electroplating | Electroplating 4 ^149^ |
| Electroplating – site 5 | Electroplating | A7^150^, Electroplating 5 ^149^ |
| Foam Insulation - site 1 | Foam Insulation | Foam insulation 1 ^149^ |
| Foam Insulation - site 2 | Foam Insulation | B8^148^, Foam insulation ^149^ |
| Manufacturing - site 1 | Manufacturing | Inorganic chemicals ^149^ |
| Manufacturing - site 2 | Manufacturing | Organic chemicals ^149^ |
| Manufacturing - site 3 | Manufacturing | Synthetic rubber ^149^ |
| Manufacturing - site 4 | Manufacturing | – |
| Manufacturing - site 5 | Manufacturing | A1^150^, Printing ink ^149^ |
| Manufacturing - site 6 | Manufacturing | B7^148^, Plastics manufacturing ^149^ |
| Petrochemical - site 1 | Petrochemical | A3^150^, Petrochemical 1 ^149^ |
| Petrochemical - site 2 | Petrochemical | B5^148^, Petrochemical 2 ^149^ |
| Commercial Truck washing - site 1 | Truck Washing | Commercial truck washing 1 ^149^ |
| Commercial Truck washing - site 2 | Truck Washing | Commercial truck washing 2 ^149^ |
| Commercial Truck washing - site 3 | Truck Washing | Commercial truck washing 3 ^149^ |
| Untreated influent - site 1 | WWTP | Untreated influent 1 ^149^ |
| Treated effluent - site 1 | WWTP | Treated effluent 1 ^149^ |
| Untreated influent - site 2 | WWTP | A4^150^, Untreated influent 2 ^149^ |
| Treated effluent - site 2 | WWTP | A5^150^, Treated effluent 2 ^149^ |
| Other - site 1 | Other | A2^150^, Automotive ^149^ |
| Other - site 8 | Other | Refrigeration ^149^ |
| Other - site 3 | Other | Cannabis production ^149^ |
| Other - site 4 | Other | A8^150^, Cosmetics ^149^ |
| Other - site 5 | Other | Healthcare linen cleaning ^149^ |
| Other - site 6 | Other | A6^150^, Personal care products ^149^ |
| Other - site 7 | Other | B4^148^, Plastics recycling ^149^ |
| Other - site 8 | Other | Refrigeration ^149^ |
| Other - site 9 | Other | Industrial storm discharge ^149^ |
| Other - site 10 | Other | Work uniform cleaning ^149^ |

# References

[1] M. T. Anaraki, D. H. Lysak, R. Soong, M. J. Simpson, M. Spraul, W. Bermel, H. Heumann, M. Gundy, H. Boenisch, A. J. Simpson, *The Analyst* **2020**, *145*, 5787.

[2] S. Bürgmayr, J. Tanner, W. Batchelor, A. F. A. Hoadley, *Nord Pulp Pap Res J* **2023**, *38*, 181.

[3] G. N. Baroi, H. N. Gavala, P. Westermann, I. V. Skiadas, *Ind Crops Prod* **2017**, *104*, 68.

[4] N. R. Saha, I. Roy, G. Sarkar, A. Bhattacharyya, R. Das, D. Rana, R. Banerjee, A. K. Paul, R. Mishra, D. Chattopadhyay, *Carbohydr Polym* **2018**, *187*, 8.

[5] K. H. Kim, X. Jin, A. Ji, A. Aui, M. Mba-Wright, C.-J. Yoo, J.-W. Choi, J.-M. Ha, C. S. Kim, C. G. Yoo, J. W. Choi, *Waste Manag* **2022**, *144*, 41.

[6] S. S. Bhagwat, Y. Li, Y. R. Cortés-Peña, E. C. Brace, T. A. Martin, H. Zhao, J. S. Guest, *ACS Sustain Chem Eng* **2021**, *9*, 16659.

[7] B. S. Giri, A. A. Juwarkar, D. B. Satpute, S. N. Mudliar, R. A. Pandey, *Appl Biochem Biotechnol* **2012**, *167*, 1744.

[8] Y. Qiao, X. Wang, H. Dai, *RSC Adv.* **2021**, *11*, 30961.

[9] C. Marcello, A. A. Salam, *Carbohydr Polym* **2023**, *299*, 120194.

[10] O. Botalova, J. Schwarzbauer, *Water Air Soil Pollut* **2011**, *221*, 77–98.

[11] Y. Wang, C. Marcello, N. Sawant, A. Salam, S. Abubakr, D. Qi, K. Li, *Cellulose* **2023**, *30*, 1957.

[12] R. Sommerville, J. Shaw-Stewart, V. Goodship, N. Rowson, E. A. Kendrick, *Sustain Mater Technol* **2020**, *25*, e00197.

[13] D. C. Hayes, S. A. Langdon, R. M. Spilker, R. Agrawal, *ACS Appl Energy Mater* **2024**, *7*, 885.

[14] L. E. Vanatta, D. E. Coleman, A. Woodruff, *J Chromatogr A* **2003**, *997*, 269.

[15] T. Liu, R. Guo, Y. Fu, J. Zhao, H. Ning, Z. Fang, Z. Liang, X. Wei, R. Yao, J. Peng, *Langmuir* **2022**, *38*, 9955.

[16] H.-J. Lee, S.-I. Na, *Mater Lett* **2022**, *312*, 131651.

[17] S. He, Y.-A. Shen, B. Xiong, F. Huo, J. Li, J. Ge, Z. Pan, W. Li, C. Hu, H. Nishikawa, *J Mater Res Technol* **2022**, *21*, 2352.

[18] S. Kim, J. Yun, J. Cho, H. Choi, Y. S. Shin, H. Jeong, J. C. Jung, *Mol Catal* **2022**, *532*, 112721.

[19] H. Li, E. A. Oraby, J. J. Eksteen, *Resour Conserv Recycl* **2022**, *187*, 106631.

[20] Y.-T. Lin, M.-L. Wang, C.-F. Hsu, W.-P. Dow, S.-M. Lin, J.-J. Yang, *J Electrochem Soc* **2013**, *160*, D3149.

[21] X. He, J. Yang, Q. Zou, Z. Hu, L. Wu, *J Electrochem Soc* **2022**, *169*, 022502.

[22] G. A. G. Pedroza, C. A. C. De Souza, M. D. De Jesus, L. R. P. De Andrade Lima, D. V. Ribeiro, *Surf Coat Technol* **2014**, *258*, 232.

[23] Q. Yang, F. Yuan, Y. Ma, K. Shi, G. Yang, J. Zhu, *Powder Technol* **2020**, *360*, 444.

[24] L. Zhu, Y. Li, G. A. Zhu, *Actuators B Chem* **2004**, *98*, 115.

[25] J. Sudagar, J. Lian, W. Sha, *J Alloys Compd* **2013**, *571*, 183.

[26] T. Yanai, T. Akiyoshi, T. Yamaguchi, K. Takashima, T. Morimura, M. Nakano, H. Fukunaga, *AIP Adv* **2018**, *8*, 056106.

[27] L. Jinhui, W. Ying, W. Yudong, G. Yang, Y. Yang, W. Ruixiang, *Front Chem* **2021**, *9*, 592407.

[28] J. Zhang, Z. Peng, R. Tian, H. Tang, L. Ye, M. Rao, G. Li, *J Therm Anal Calorim* **2023**, *148*, 10335.

[29] G. M. Zarkadas, A. Stergiou, G. Papanastasiou, *Electrochimica Acta* **2005**, *50*, 5022.

[30] K. Kaewket, K. Ngamchuea, *RSC Adv* **2023**, *13*, 33210.

[31] C. Lin, J. Hu, J. Zhang,P. Yang, X. Kong, G. Han, Q. Li, M. An, *Surf Interfaces* **2021**, *22*, 100804.

[32] Yu. D. Gamburg, M. Yu. Grosheva, S. Biallozor, M. Hass, *Surf Coat Technol* **2002**, *150*, 95.

[33] M. A. M. Ibrahim, R. M. Al Radadi, *Int J Electrochem Sci* **2015**, *10*, 4946.

[34] I. V. Antihovich, N. M. Ablazhey, A. A. Chernik, I. M. Zharsky, *Procedia Chem* **2014**, *10*, 373.

[35] L. Philippe, C. Heiss, J. Michler, *Chem Mater* **2008**, *20*, 3377.

[36] T. Tsuru, S. Kobayashi, T. Akiyama, H. Fukushima, S. K. Gogia, R. Kammel, *J Appl Electrochem* **1997**, *27*, 209.

[37] P. S. D. Brito, S. Patrício, L. F. Rodrigues, C. A. C. Sequeira, *Surf Coat Technol* **2012**, *206*, 3036.

[38] H. Zhang, R. Cui, *Membranes* **2024**, *14*, 240.

[39] S. N. Srimathi, S. M. Mayanna, *Mater Chem Phys* **1984**, *11*, 351.

[40] H. Liang, Y. Li, X. Zhao, C. Gao, H. Zhang, Z. Geng,D. She, *J Hazard Mater* **2022**, *437*, 129345.

[41] O. Aras, E. Baydır, B. Akman, *Appl Phys A* **2022**, *128*, 176.

[42] L. Ricci, M.-F. Tseng, M. Tao, *Sol Energy Mater Sol Cells* **2022**, *240*, 111689.

[43] S. Syamsuir, B. Soegijono, S. D. Yudanto, B. Basori, M. K. Ajiriyanto, D. Nanto, F. B. Susetyo, *Int J Eng* **2023**, *36*, 1193.

[44] C. D. Gu, Y. H. You, Y. L. Yu, S. X. Qu, J. P. Tu, *Surf Coat Technol* **2011**, *205*, 4928.

[45] M. Miyake, H. Fujii, T. Hirato, *Surf Coat Technol* **2015**, *277*, 160.

[46] Q. Jaussaud, I. M. Ogbu, G. G. Pawar, E. Grau, F. Robert, T. Vidil, Y. Landais, H. Cramail, *Chem Sci* **2024**, *15*, 13475.

[47] M. Ates, S. Karadag, A. A. Eker, B. Eker, *Polym Int* **2022**, *71*, 1157.

[48] S. B. Murmu, *Clean Eng Technol* **2022**, *8*, 100513.

[49] M. A. Ansari, P. Somdee, K. Marossy, *J Polym Res* **2021**, *28*, 184.

[50] J. Deyo, Butyric Acid*. In *Encyclopedia of Toxicology*; Elsevier, **2005**; pp 368–370.

[51] T. Rokkonen, P. Willberg-Keyriläinen, J. Ropponen, T. Malm, *Polymers* **2021**, *13*, 2416. h

[52] Q. Zhao, M. Qu, Y. Song, S. Li, W. Zhao, P. Tang, Y. Bin, H. Wang, *J Mater Sci* **2022**, *57* , 12147.

[53] D. Sridaeng, W. Jitaree, P. Thiampanya, N.Chantarasiri, *E-Polym* **2016**, *16*, 265.

[54] M. Modesti, N. Baldoin, F. Simioni, *Eur Polym J* **1998**, *34*, 1233.

[55] J. Yu, L. Sun, L. Ding, Y. Cao, X. Liu, Y. Ren, Y. Li, *Polym Degrad Stab* **2024**, *227*, 110892.

[56] M. Nofar, C. B. Park, *Prog Polym Sci.* **2014**, *39*, 1721.

[57] M. Liu, Z. Gong, G. Wang, X. Liu, Y. Hou, G. Tang, *Polym Degrad Stab* **2024**, *219*, 110605.

[58] X. Zhi, X. Bian, J. Yu, X. Xiao, B. Duan, F. Huang, Z. Jiang, G. Zhou, N. Ma, *Agric Water Manag* **2024**, *296*, 108797.

[59] M. Stanzione, V. Russo, M. Oliviero, L. Verdolotti, A. Sorrentino, M. Di Serio, R. Tesser, S. Iannace, M. Lavorgna, *Polymer* **2018**, *149*, 134.

[60] K. Kizuka, S.-I. Inoue, *Open J Org Polym Mater* **2016**, *06*, 38.

[61] C. Guo, Y. Huo, Q. Zhang, K. Wan, G. Yang, Z. Liu, F. Peng, *Nanomaterials* **2023**, *13*, 2318.

[62] Ł. Krysztofik, B. Osowiecka, B. Liszyńska, J. Zieliński, M. Zakarzecki, *Polimery* **2022**, *67*, 271.

[63] R. K. Saxena, P. Anand, S. Saran, J. Isar, L. Agarwal, *Indian J Microbiol* **2010**, *50*, 2.

[64] Z. Freixa, Rhodium-Catalyzed Methanol Carbonylation. In *Reference Module in Chemistry, Molecular Sciences and Chemical Engineering*; Elsevier, **2019**; p B9780124095472110650.

[65] W. Young, L. DeJager, *Food Addit Contam Part A* **2018**, *35*, 2309.

[66] H. Robles, Formic Acid. In *Encyclopedia of Toxicology*; Elsevier, **2005**; pp 378–380.

[67] X. Zhou, M. Zha, J. Cao, H. Yan, X. Feng, D. Chen, C. Yang, *ACS Sustain Chem Eng* **2021**, *9*, 10948.

[68] I. Goldberg, J. S. Rokem, Organic and Fatty Acid Production, Microbial. In *Encyclopedia of Microbiology*; Elsevier, **2009**; pp 421–442.

[69] F. A. McArdle, *The Analyst* **1998**, *123*, 1757.

[70] W. E. Luttrell, A. L. LaGrow, *J Chem Health Saf* **2014**, *21*, 29.

[71] R. F. Dye, *Korean J Chem Eng* **2001**, *18*, 571.

[72] J. Hugenholtz, E. J. Smid, *Curr Opin Biotechnol* **2002**, *13*, 497.

[73] H. Yakan, S. Cakmak, H. Kutuk, S. Yenigun, T. Ozen, *Res Chem Intermed* **2020**, *46*, 2767.

[74] S. Mallakpour, V. Behranvand, *Des Monomers Polym* **2016**, *19*, 283.

[75] K. S. Hayes, *Appl Catal Gen* **2001**, *22*, 187.

[76] F. A. Castillo Martinez, E. M. Balciunas, J. M. Salgado, J. M. Domínguez González, A. Converti, R. P. D. S. Oliveira, *Trends Food Sci Technol* **2013**, *30*, 70.

[77] C. S. Hsu, P. R. Robinson, *Petroleum Science and Technology: Downstream*; Springer Nature Switzerland: Cham, **2024**.

[78] O.-W. Achaw, E. Danso-Boateng, Soaps and Detergents. In *Chemical and Process Industries*; Springer International Publishing: Cham, **2021**; pp 1–37.

[79] H. C. Genuino, N. N. Opembe, E. C. Njagi, S. McClain, S. L. *J Ind Eng Chem* **2012**, *18*, 1529.

[80] N. Musee, P. Ngwenya, L. K. Motaung, K. Moshuhla, P. Nomngongo, *Environ Chem Ecotoxicol* **2023**, *5*, 62.

[81] C. C. Viggi, D. Dionisi, A. Miccheli, M. Valerio, M. Majone, *Water Res* **2010**, *44*, 3393.

[82] Y. Zhu, K. Xiao, Y. Zhou, W. Yu, S. Tao, C. Le, D. Lu, Z. Yu, S. Liang, J. Hu, H. Hou, B. Liu, J. Yang, *Water Res* **2020**, *175*, 115645.

[83] Y. Dong, L. Ma, Y. Peng, Z. Hu, Z. Xu, X. Jiang, R. Cheng, H. Chen, Q. Yang, *J Water Process Eng* **2023**, *54*, 103961.

[84] S. Navalon, M. Alvaro, H. Garcia, *Environ Technol* **2011**, *32*, 295.

[85] S. Longo, E. Katsou, S. Malamis, N. Frison, D. Renzi, F. Fatone, *Bioresour Technol* **2015**, *175*, 436.

[86] U. Kotowska, K. Bieganska, V. A. Isidorov, *Pol J Environ Stud* **2012**, *21*, 129.

[87] M.-F. Dignac, P. Ginestet, D. Rybacki, A. Bruchet, V. Urbain, P. Scribe, *Water Res* **2000**, *34*, 4185.

[88] L. Wang, M. Min, Y. Li, P. Chen, Y. Chen, Y. Liu, Y. Wang, R. Ruan, *Appl Biochem Biotechnol* **2010**, *162*, 1174.

[89] L. Pan, L. Tian, L. Wang, *J Environ Chem Eng* **2024**, *12*, 113081.

[90] L. Zhao, J. Pan, Y. Ding, S. Cai, T. Cai, L. Chen, X.-M. Ji, *Int J Biol Macromol* **2023**, *226*, 1523.

[91] Q. Yang, H. Li, D. Wang, X. Zhang, X. Guo, S. Pu, R. Guo, J. Chen, *Appl Energy* **2020**, *276*, 115502.

[92] M. Ilyas, W. Ahmad, H. Khan, *Desalination Water Treat* **2022**, *256*, 194.

[93] V. Kumar, M. Sankaranarayanan, K. Jae, M. Durgapal, S. Ashok, Y. Ko, R. Sarkar, S. Park, *Appl Microbiol Biotechnol* **2012**, *96*, 373.

[94] D. Song, Y.-G. Yoon, C.-J. Lee, *Chem Eng Res Des* **2017**, *123*, 268.

[95] V. M. Craddock, *Br. J. Cancer* **1978**, *37*, 322.

[96] P. Khare, N. Kumar, K. M. Kumari, S. S. Srivastava, *Rev Geophys* **1999**, *37*, 227.

[97] M. Alahiane, R. Oukhrib, A. Berisha, Y. A. Albrimi, R. A. Akbour, H. A. Oualid, H. Bourzi, A. Assabbane, A. Nahlé, M. Hamdani, *J Mol Liq* **2021**, *328*, 115413.

[98] S. M. Prasanth, P. S. Kumar, S. Harish, M. Rishikesh, S. Nanda, D.-V. N. Vo, *Chemosphere* **2021**, *280*, 130723.

[99] N. Joy, J. Jayaprabakar, M. Anish, J. Giri, E. Makki, T. Sathish, *Case Stud Therm Eng* **2024**, *60*, 104656.

[100] A. Saha, S. Sengupta, A. Virmani, A. Kumar, *J Chem Sci* **2022**, *134*, 98.

[101] D. Bir, K. Tutin, *J Chromatogr Sci* **2002**, *40* 337.

[102] C. Yangin-Gomec, G. Engiz, *Heliyon* **2021**, *7*, e06296.

[103] A. F. A. Nabil, I. E. Putri, W. Saputro, E. A. Saputro, Mass Balance Analysis of Carbon Black Production from Waste Polyethylene Terephthalate (PET). In *Nusantara Science and Technology Proceedings*; Galaxy Science, **2022**.

[104] R. J. Stoklosa, R. J. Latona, B. W. Berger, M. P. Timko, A. V. Shlanta, M. R. Himes, *ACS Sustain Resour Manag* **2024**, *1*, 939.

[105] B. S. Audu, P. C. Ofojekwu, A. Ujah, M. N. O. Ajima, *J Phytopharm.* **2014**, *3*, 35.

[106] L. Serventi, G. A. Flores, G. Cusumano, D. Barbaro, B. Tirillini, R. Venanzoni, P. Angelini, A. Acquaviva, S. C. Di Simone, G. Orlando, G. Zengin, L. Menghini, C. Ferrante, *Antioxidants* **2023**, *12*, 219.

[107] Y. Jiang, Y. Sun, D. Zheng, C. Han, K. Cao, L. Xu, S. Liu, Y. Cao, N. Feng, *Sci Rep* **2021**, *11*, 14476.

[108] G. G. Kanyairita, D. G. Mortley, M. Boersma, W. E. Collier, *Separations* **2024**, *11*, 106.

[109] I. B. Gunnarsson, M. Kuglarz, D. Karakashev, I. Angelidaki, *Bioresour Technol* **2015**, *182*, 58.

[110] B. Heldreth, W. F. Bergfeld, D. V. Belsito, R. A. Hill, C. D. Klaassen, D. Liebler, J. G. Marks, R. C. Shank, T. J. Slaga, P. W. Snyder, F. A. Andersen, *Int J Toxicol* **2012**, *31*, 112S.

[111] P. Song, X. Zhang, S. Wang, W. Xu, F. Wei, *Front Bioeng Biotechnol* **2023**, *11*, 1283129.

[112] A. Del Olmo, J. Calzada, M. Nuñez, *Crit Rev Food Sci Nutr* **2017**, *57*, 3084.

[113] L. Jiang, H. Fu, H. K. Yang, W. Xu, J. Wang, S.-T. Yang, *Biotechnol Adv* **2018**, *36*, 2101.

[114] R. I. Peirano, V. Achterberg, H.-J. Düsing, M. Akhiani, U. Koop, S. Jaspers, A. Krüger, H. Schwengler, T. Hamann, H. Wenck, F. Stäb, S. Gallinat, T. Blatt, *J Cosmet Dermatol* **2011**, *10*, 273.

[115] W. Johnson, B. Heldreth, W. F. Bergfeld, D. V. Belsito, R. A. Hill, C. D. Klaassen, D. C. Liebler, J. G. Marks, R. C. Shank, T. J. Slaga, P. W. Snyder, F. A. Andersen, *Int J Toxicol* **2016**, *35*, 41S.

[116] S. Yang, L. Long, D. Li, J. Zhang, S. Jin, F. Wang, J. Chen, *Aging Cell* **2015**, *14*, 1024.

[117] A. Komesu, J. A. R. D. Oliveira, L. H. D. S. Martins, M. R. Wolf Maciel, R. Maciel Filho, *BioResources* **2017**, *12*, 4364.

[118] Z. Zhong, G. Li, B. Zhu, Z. Luo, *J Liq Chromatogr Relat Technol* **2012**, *35*, 1719.

[119] R. Gonzalez-Garcia, T. McCubbin, L. Navone, C. Stowers, L. Nielsen, E. Marcellin, *Fermentation* **2017**, *3*, 21.

[120] F. Grafe, W. Wohlrab, R. H. Neubert, M. Brandsch, *Eur J Pharm Biopharm* **2004**, *57*, 337.

[121] Y. Tao, C. Bu, L. Zou, Y. Hu, Z.-J. Zheng, J. A. Ouyang, *Biotechnol Biofuels* **2021**, *14*, 216.

[122] M.-K. Zinn, D. Bockmühl, *BMC Microbiol* **2020**, *20*, 265.

[123] S. Ahmadi, D. Winter, *Anal Chem* **2018**, *90*, 6594.

[124] M. Rekaby, H. M El-Hennawi, A. A. Shahin, A. A. Ragheb, *Carbohydr Polym* **2013**, *98*, 1540.

[125] T. Attarbachi, M. D. Kingsley, V. Spallina, *Fuel* **2023**, *340*, 127485.

[126] O. Troynikov, C. Watson, A. Jadhav, N. Nawaz, R. Kettlewell, *J Environ Manage* **2016**, *182*, 252.

[127] A. C. Kogawa, B. G. Cernic, L. G. D. Do Couto, H. R. N. Salgado, *Saudi Pharm J* **2017**, *25*, 934.

[128] S. G. Chu, Y. J. Chang, J. Y. Ryu, J. S. Lee, K. Y. Choi, H. Y. Chung, B. C. Cho, J. D. Yang, *In Vivo* **2022**, *36*, 2714.

[129] L. Maria, N. Jamil, M. Zaheer, N. Hussain, M. Naeem, N. Ahsan, *J Water Chem Technol* **2020**, *42*, 465.

[130] M. M. Sy, E. Garcia-Hidalgo, C. Jung, O. Lindtner, N. Von Goetz, M. Greiner, *Food Chem Toxicol* **2020**, *140*, 111320.

[131] U. Klaschka, *Environ Sci Eur* **2016**, *28*, 8.

[132] S. Wang, J. Li, X. Li, Y. Tu, *Fundam Res* **2024**, S2667325824002516.

[133] M. Y. Kariduraganavar, A. A. Kittur, R. R. Kamble, Polymer Synthesis and Processing. In *Natural and Synthetic Biomedical Polymers*; Elsevier, **2014**; pp 1–31.

[134] M. T. Chin, T. Diao, *ACS Catal* **2024**, *14*, 12437.

[135] E. Feghali, L. Tauk, P. Ortiz, K. Vanbroekhoven, W. Eevers, *Polym Degrad Stab* **2020**, *179*, 109241.

[136] Y. Dong, J. Wang, Y. Yang, Q. Wang, X. Zhang, H. Hu, J. Zhu, *Polym Degrad Stab* **2022**, *202*, 110010.

[137] A.-C. Enache, I. Grecu, P. Samoila, *Materials* **2024**, *17*, 2991.

[138] L. Shen, E. Worrell, Plastic Recycling. In *Handbook of Recycling*; Elsevier, **2024**; pp 497–510.

[139] P. Sambyal, P. Najmi, D. Sharma, E. Khoshbakhti, H. Hosseini, A. S. Milani, M. Arjmand, *Can J Chem Eng* **2024**, cjce.25531.

[140] M. J. Boel, H. Wang, A. Al Farra, L. Megido, J. M. González-LaFuente, N. R. Shiju, *React Chem Eng* **2024**, *9*, 1014.

[141] B. L. Kaul, Plastic Additives for Fire Safety with Weight Reduction, Thermal Stability in Processing and Waste Management Morpholino-Poly(Piperazinyl-Morpholinyl-Triazins)*. **2021**.

[142] A. Cabanes, A.; Fullana, *Sci Total Environ* **2021**, *758*, 144066.

[143] M. Lache, C. Kappelhoff, J. Seiler, A. Bardow, *Energy Technol* **2023**, *11*, 2201158.

[144] Y. Zhao, X. Zhang, X. Xu, S. Zhang, *J Mol Liq* **2020**, *319*, 114360.

[145] V. Na Nagara, D. Sarkar, M. Boufadel, R. Datta, *Chemosphere* **2023**, *331*, 138779.

[146] I. Nicander, I. Rantanen, B. L. Rozell, E. Söderling, S. Ollmar, *Skin Res Technol* **2003**, *9*, 50.

[147] M. Chirac, S. Cambos, J. Guilbot, C. Nawrocki, R. Marchand, A. Roso, *J Surfactants Deterg* **2024**, *27*, 753.

[148] E. A. Oliveira Pereira, L. M. Labine, S. Kleywegt, K. J. Jobst, A. J. Simpson, M. J. Simpson, *Environ Res* **2024**, *241*, 117547.

[149] K. L. Steeves, M. J. Bissram, S. Kleywegt, D. Stevens, F. L. Dorman, A. J. Simpson, M. J. Simpson, L. S. Cahill, K. J. Jobst, *Environ Int* **2023**, *171*, 107634.

[150] L. M. Labine, E. A. O. Pereira, S. Kleywegt, K. J. Jobst, A. J. Simpson, M. J. Simpson, *Environ Res* **2023**, *234*, 116512.
